# BMP and STRA8 act collaboratively to ensure correct mitotic-to-meiotic transition in the fetal mouse ovary

**DOI:** 10.1242/dev.204227

**Published:** 2025-02-07

**Authors:** Fiona K. M. Cheung, Chun-Wei Allen Feng, Clare Crisp, Yuji Mishina, Cassy M. Spiller, Josephine Bowles

**Affiliations:** ^1^ School of Biomedical Sciences, The University of Queensland, Brisbane, Queensland 4072, Australia; ^2^ School of Dentistry, University of Michigan, Ann Arbor, MI 48109, USA; ^3^ Institute for Molecular Bioscience, The University of Queensland, Brisbane, Queensland 4072, Australia

**Keywords:** BMP signalling, STRA8, Fetal ovary, Fetal ovarian germ cell, Mitosis-to-meiosis transition, Mammalian reproduction

## Abstract

A successful mitosis-to-meiosis transition in germ cells is essential for fertility in sexually reproducing organisms. In mice and humans, it has been established that expression of STRA8 is crucial for meiotic onset in both sexes. Here, we show that BMP signalling is also essential, not for STRA8 induction but for correct meiotic progression in female mouse fetal germ cells. Largely in agreement with evidence from primordial germ cell-like cells (PGCLCs) *in vitro*, germ cell-specific deletion of BMP receptor 1A (BMPR1A; ALK3) caused aberrant retention of pluripotency marker OCT4 and meiotic progression was compromised; however, the timely onset of *Stra8* and STRA8 expression was unaffected. Comparing the transcriptomes of *Bmpr1a*-cKO and *Stra8*-null models, we reveal interplay between the effects of BMP signalling and STRA8 function. Our results verify a role for BMP signalling in instructing germ cell meiosis in female mice *in vivo*, and shed light on the regulatory mechanisms underlying fetal germ cell development.

## Teaser

**Summary:** We demonstrate, *in vivo*, a requirement for BMP signalling in fetal ovarian germ cell meiosis, and reveal that timely mitosis-to-meiosis transition requires both STRA8 and BMP signalling.

## INTRODUCTION

In most animals, the sole means by which genetic material is transferred from one generation to the next is via haploid gametes, and their production is crucially dependent on the process of meiosis. Although the chromosomal events of meiosis are relatively conserved from yeasts to humans, the molecular regulators of the decision to switch from mitosis to meiosis are less conserved; perhaps for this reason, meiotic initiation has been under-studied (Kimble, 2011).

Primordial germ cells (PGCs) are specified in the mouse epiblast at embryonic (E) day ∼7.25 through the coordinated signalling activities of different factors, including BMP2, BMP4, BMP8b, and WNT3a (Lawson et al., 1999; Ohinata et al., 2009; Saitou et al., 2002; Ying et al., 2000, 2001). Nascent PGCs maintain or upregulate pluripotency marker expression [e.g. *Oct4* (*Pou5f1*) and *Sox2*], repress somatic genes and begin to express germ cell specific genes. PGCs migrate to and colonise the embryonic gonads from ∼E10.5, at which time genome-wide epigenetic reprogramming occurs (Hajkova, 2010; Hajkova et al., 2002). Once in the gonads, PGC sexual fate is determined not by their genetic sex but by the signals received from the gonadal soma (McLaren, 1995, 2003). Gonadal somatic cues direct ovarian germ cells to enter meiosis during fetal life, but testicular germ cells avoid meiotic entry until after birth. Despite this knowledge, we still lack a comprehensive understanding of soma-to-germ cell signalling during fetal gonad development.

Evidence exists that the signalling molecule retinoic acid (RA) directly induces mouse fetal ovarian germ cells to initiate *Stra8* (stimulated by retinoic acid gene 8) expression (Bowles et al., 2006, 2016; Feng et al., 2021; Koubova et al., 2006; Soh et al., 2015). *Stra8* encodes a transcription factor that is crucial for the initiation of meiotic S (synthesis) phase and for meiotic progression in both sexes. In the *Stra8*-null mouse, crucial features of meiosis, including chromosome condensation, cohesion, synapsis, and recombination, are absent (Baltus et al., 2006). Because RA levels are highest at the anterior end of the developing ovary, *Stra8* transcript and STRA8 protein are induced in an anterior-to-posterior ‘wave’ (Bowles et al., 2006; Menke et al., 2003; Feng et al. 2021). In the fetal testis, RA is cleared by the RA-degrading enzyme CYP26B1 (Bowles et al., 2006; Dokshin et al., 2013; MacLean et al., 2007; Soh et al., 2015) and, therefore, testicular germ cells are spared from meiotic entry during fetal life. Besides *Stra8*, other studies have shown that RA directly induces other key meiotic genes, including *Rec8* (Koubova et al., 2014) and *Meiosin* (Ishiguro et al., 2020).

Recent findings suggest that, at least *in vitro*, another signalling molecule, bone morphogenetic protein (BMP), is necessary for XX germ cell development (Miyauchi et al., 2017). BMPs are TGFβ superfamily signalling ligands; they transduce signal via binding to type I and type II transmembrane serine/threonine kinase receptors (Mueller and Nickel, 2012). BMPs have numerous roles in germ cell specification (BMP2, BMP4 and BMP8b) (Lawson et al., 1999; Ying et al., 2000, 2001), migration (BMP4) (Dudley et al., 2007), and postnatal oocyte development (GDF9 and BMP15) (Dong et al., 1996; Yan et al., 2001). In a landmark study, primordial germ cell-like cells (PGCLCs), generated *in vitro* from mouse embryonic stem cells and cultured without somatic cells (Ohta et al., 2017), were studied to delineate the signalling activity necessary to drive mitosis-to-meiosis transition, meiotic progression, and oogenesis (Miyauchi et al., 2017). It was concluded that RA and BMP work synergistically to promote oogenic fate (Miyauchi et al., 2017) and the transcription factor ZGLP1 was subsequently identified as a key effector of BMP signalling in PGCLCs (Nagaoka et al., 2020). Despite this progress, it remains unclear whether BMP signalling plays a crucial role *in vivo* and, if so, at which step(s) of ovarian germ cell development it is required.

Investigating whether BMPs act directly on gonadal germ cells to instruct female-specific development is complicated because: (1) BMP signalling is required for earlier PGC specification and proliferation (Lawson et al., 1999; Ross et al., 2007; Ying et al., 2000, 2001); (2) several BMP ligands (BMP2, BMP4 and BMP5) are expected to be present in the E11.5 fetal ovary (Jameson et al., 2012; Ross et al., 2007; Yao et al., 2004); (3) BMP2 likely also supports correct fetal ovarian somatic cell development (Kashimada et al., 2011; Yao et al., 2004); and (4) BMPs signal through receptors shared with other TGFβ family members. Given these considerations, we investigated the role of BMP in control of ovarian germ cell fate using two approaches: broad chemical inhibition of BMP signalling in *ex vivo* culture and germ cell-specific deletion of the gene encoding the BMP receptor 1A (BMPR1A) *in vivo*.

## RESULTS

### Antagonising canonical BMP signalling impacts expression of *Sycp3* and *Oct4* in *ex vivo* UGR cultures

To investigate the potential role of BMP signalling in meiotic onset in fetal ovarian germ cells, we inhibited BMP signalling in urogenital ridges (UGRs) cultured *ex vivo*. LDN193189, a small molecule inhibitor of BMPR1A (ALK3) and ACVR1 (ALK2) (Cuny et al., 2008), specifically inhibits canonical BMP signalling by blocking SMAD1/5/8 phosphorylation. UGRs were dissected from E11.5 C57BL/6 embryos and individually cultured *ex vivo* for 24, 48 or 72 h in hanging drops with or without LDN193189 (500 nM) (Fig. S1A). Antagonism of BMPR1A and ACVR1 led to a significant upregulation of *Bmp2* expression in the 24 h group (Fig. S1B), possibly reflecting a feedback mechanism by the gonadal somatic cells to compensate for reduced BMP signalling.

Treatment with LDN193189 did not adversely affect the onset of *Stra8* expression; rather, *Stra8* was elevated in 48- and 72-h treatment groups (Fig. S1C). This finding could suggest that disruption to BMP signalling enhances *Stra8* expression. It seems more likely, however, that this result reflects delayed germ cell progression through meiosis, because STRA8 downregulates its own expression as meiosis proceeds (Soh et al., 2015). Consistent with the latter possibility, *Sycp3* expression, a marker of meiotic progression, was significantly lower in 72 h treated samples compared to controls (Fig. S1C). Meiotic entry coincides with downregulation of pluripotency marker *Oct4* (Bullejos and Koopman, 2004). We found that blocking of BMP signalling in UGRs did not affect *Oct4* expression in 24 or 48 h groups, though expression was abnormally maintained at 72 h (Fig. S1C). These *ex vivo* culture studies suggest that BMP signalling is likely required for efficient meiotic progression and not necessarily for meiotic onset as perturbations to *Sycp3* and *Oct4* only occurred after 72 h.

### Germ cell-specific knockout of *Bmpr1a* is effective and specific in mouse fetal ovaries

Next, we used a genetic deletion approach to investigate the *in vivo* role for BMP signalling and to test whether its effects are direct or indirect on the germ cells. BMP2 is a likely candidate for directing fetal ovarian germ cell development due to its female-specific gene expression (Yao et al., 2004). The *Bmp2*-null mutant is embryonic lethal at E10.5 (Zhang and Bradley, 1996), and BMP2 likely plays important roles in ovarian soma development (Bayne et al., 2016; Kashimada et al., 2011; Yao et al., 2004). Furthermore, *Bmp4* and *Bmp5* expression may also contribute to signalling in the fetal ovary (Jameson et al., 2012). To circumvent these problems, we deleted the type I BMP receptor BMPR1A (used by BMP2, BMP4 and BMP5) specifically in gonadal germ cells (*Bmpr1a^ΔPGC^*) using *Bmpr1a^tm2.1Bhr^* (*Bmpr1a^fl/fl^*) (Mishina et al., 2002) and *Oct4-CreERT2* (Greder et al., 2012) mouse lines.

**Fig. 1.**
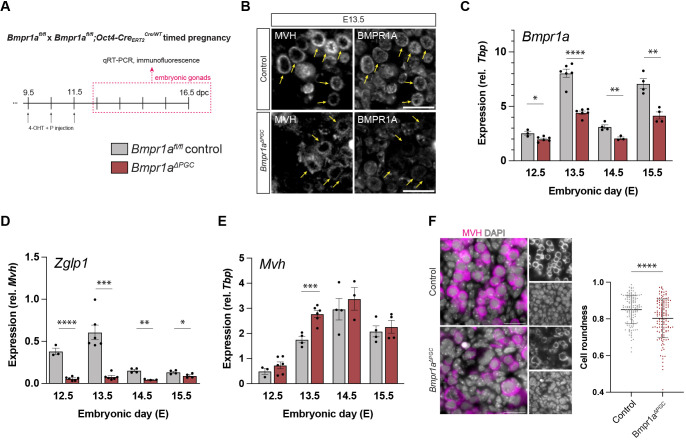
**Germ cell-specific knockout of *Bmpr1a* substantially reduced expression of downstream effector *Zglp1* but did not affect germ cell survival.** (A) *Bmpr1a^fl/fl^* females were time-mated with *Bmpr1a^fl/fl^;Oct4-Cre^Cre/WT^* males; CRE-dependent recombination of *Bmpr1a* in the embryos was induced by intraperitoneal administration of 4-hyroxytamoxifen (4-OHT) and progesterone (P) to the dams at 9.5, 10.5, and 11.5 dpc. Embryos were harvested at E12.5-E16.5 for gene and protein expression analysis. (B) Immunostaining for BMPR1A and MVH in E13.5 embryos confirmed successful depletion of BMPR1A in germ cells*.* (C,D) qRT-PCR showed significant downregulation of (C) *Bmpr1a* and (D) *Zglp1* in *Bmpr1a^ΔPGC^* ovaries compared with the control across all timepoints analysed. (E) *Mvh* was significantly higher in the mutants at E13.5 only. (F) *Bmpr1a^ΔPGC^* germ cells are less round compared to control germ cells. *P<0.05, **P<0.01, ***P<0.001, ****P<0.0001 (*n*≥4, unpaired *t*-test; data are mean±s.e.m. for qRT-PCR and mean±s.d. for cell roundness). Cytoplasmic MVH (magenta) marks germ cells. Scale bars: 20 μm.

*Bmpr1a^fl/fl^* females were time-mated with *Bmpr1a^fl/fl^;Oct4-Cre^Cre/WT^* males, and injected with 4-hydroxytamoxifen at 9.5, 10.5, and 11.5 days post coitum (dpc) to induce CRE-dependent deletion of *Bmpr1a* in embryonic germ cells. *Bmpr1a^ΔPGC^* and *Bmpr1a^fl/fl^* control embryos were harvested from E12.5 to E16.5 (Fig. 1A). Successful deletion was confirmed by the loss of BMPR1A immunostaining in MVH(DDX4)^+^ germ cells, but not the surrounding gonadal somatic cells, in E13.5 ovaries (Fig. 1B). Additionally, we confirmed loss of *Zglp1* expression, a BMP target in germ cells, in MACS-enriched germ cell populations (Fig. S2).

In *Bmpr1a^ΔPGC^* fetal ovaries, expression of *Bmpr1a* was significantly reduced compared to the control at all timepoints analysed (Fig. 1C). Similarly, *Zglp1* expression was significantly reduced compared to the control from E12.5-E15.5 (Fig. 1D), confirming *Zglp1* as a downstream target of BMPR1A-mediated BMP signalling in mouse ovarian germ cells *in vivo*. Contrary to observations in PGCLCs, and after ubiquitous inhibition of BMP signalling in pregnant dams (Miyauchi et al., 2017), *Mvh* expression was not diminished in our *Bmpr1a*-cKO model. *Mvh* transcription did not differ between mutant and control samples, except at E13.5, when significantly higher *Mvh* expression was found in the mutants (Fig. 1E). As *Mvh* expression (relative to *Tbp* expression) can be considered a proxy for germ cell number, this might indicate that mutant germ cells continued dividing mitotically when compared with control germ cells. We noted that germ cells deficient for BMPR1A were more irregular in shape than control germ cells (Fig. 1F); the implication of this is unknown as the survival of the cells, at least to E16.5, appeared normal.

### Germ cell-specific knockout of *Bmpr1a* did not affect *Stra8* initiation, but subcellular localisation of STRA8 protein was affected from E15.5

**Fig. 2.**
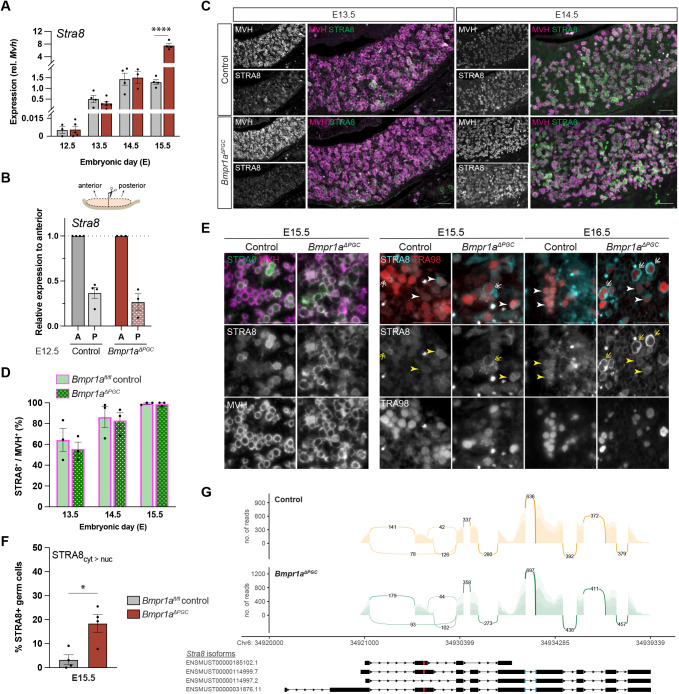
**Loss of BMP signalling did not affect temporal onset or anterior-to-posterior initiation of *Stra8* expression but resulted in increased cytoplasmic STRA8.** (A) qRT-PCR showed no difference in the onset of *Stra8* expression between control and *Bmpr1a^ΔPGC^* fetal ovaries at E12.5-E14.5, but expression level at E15.5 was substantially higher (∼5-fold) in the mutants. (B) Quantification of *Stra8* expression from anterior and posterior halves of E12.5 fetal ovaries showed the highest *Stra8* expression in the anterior half in both control and *Bmpr1a^ΔPGC^* mutant gonads. (C-E) The number of STRA8^+^ germ cells was comparable between E13.5 and E15.5 (green, STRA8; magenta, germ cell marker MVH). (E) At E15.5, most MVH^+^ germ cells expressed STRA8. Consistent across E15.5 and E16.5 control ovaries, punctate STRA8 immunostaining was found predominantly localised to the nucleus, or to both the nucleus and cytoplasm (arrowheads) of germ cells. Very few cells exhibited stronger cytoplasmic than nuclear STRA8 immunosignal (double-headed arrow). In the mutants, most *Bmpr1a^ΔPGC^* germ cells expressed both nuclear and cytoplasmic STRA8, with most cells exhibiting stronger cytoplasmic STRA8 immunosignal. MVH (magenta) and TRA98 (red) are cytoplasmic and nuclear markers for germ cells, respectively. (F) E15.5 *Bmpr1a^ΔPGC^* ovaries had significantly more germ cells with cytoplasmic-accumulated STRA8 than the control. (G) *Stra8* transcript splice junctions visualised by sashimi plot showed that both control and *Bmpr1a^ΔPGC^* ovaries expressed *Stra8* isoforms with or without the nuclear localisation sequence (NLS, represented in red within exon 3), or nuclear export sequence (NES, represented in blue across exons 6 and 7) (ENSMUST00000185102.1, ENSMUST00000114999.7, or ENSMUST00000114997.2) in similar proportions. *P<0.05, ***P<0.0001 (*n*≥4, unpaired *t*-test; data are mean±s.e.m.). Scale bars: 50 μm.

We first investigated the initial steps of germ cell differentiation. Analysis by qRT-PCR showed that loss of BMPR1A did not affect the initiation of *Stra8* expression in E12.5-E14.5 fetal ovaries (Fig. 2A). To test whether the anterior-to-posterior ‘wave’ of *Stra8* upregulation is affected (Menke et al., 2003), E12.5 fetal ovaries were bisected to compare *Stra8* expression between the anterior and posterior halves. Comparable expression between the mutants and controls indicates that loss of *Bmpr1a* did not compromise the anterior-to-posterior ‘wave’ of *Stra8* onset (Fig. 2B), suggesting that this is primarily determined by RA availability, as postulated previously (Bowles et al., 2010, 2006). At E15.5, significantly higher *Stra8* expression was observed (∼5.8-fold difference) in *Bmpr1a^ΔPGC^* ovaries (Fig. 2A), corroborating our *ex vivo* gonad culture observations (Fig. S1C), and possibly reflecting a lack of meiotic progression.

Consistent with the evidence that *Stra8* mRNA is initiated correctly, immunofluorescence staining on sectioned tissue showed the presence of STRA8^+^ germ cells at E13.5 and E14.5, with no obvious difference between mutants and controls (Fig. 2C,D, Fig. S3). At E15.5 and E16.5, however, close examination revealed an increase in cytoplasmic STRA8 in *Bmpr1a^ΔPGC^* germ cells compared to controls (Fig. 2E). STRA8 is known to shuttle between the nucleus and cytoplasm, which is important for its transcriptional activity (Tedesco et al., 2009). In control germ cells, STRA8 was found predominantly in a punctate pattern in the nucleus, with some cells being STRA8^+^ in both the nucleus and cytoplasm. In *Bmpr1a^ΔPGC^* germ cells, nuclear signal for STRA8 was observed but this was frequently accompanied by a stronger cytoplasmic signal. E15.5 mutant ovaries possessed 10 times more germ cells with a stronger STRA8 signal in the cytoplasm than nucleus (cyt>nuc) compared to the control (mutant: 17.7%, 95/937 STRA8^+^ germ cells; control: 1.8%, 17/944 STRA8^+^ germ cells) (Fig. 2F). RNA-Seq analysis (see later sections) showed that *Stra8* isoforms, with or without the N-terminal nuclear localisation signal (NLS, amino acids 28-33, depicted in red) and nuclear export sequence (amino acids 174-348, depicted in blue), were expressed in comparable ratios between E14.5 control and *Bmpr1a^ΔPGC^* ovaries (Fig. 2G), suggesting the difference in subcellular localisation is not a result of differential isoform expression. These results indicate direct BMP signalling in ovarian germ cells is dispensable for initiation of *Stra8* expression, but is necessary for the extinction of *Stra8* expression and nuclear localisation of STRA8 at later timepoints (E15.5-E16.5).

### Germ cell-specific knockout of *Bmpr1a* delayed meiotic progression *in vivo*

**Fig. 3.**
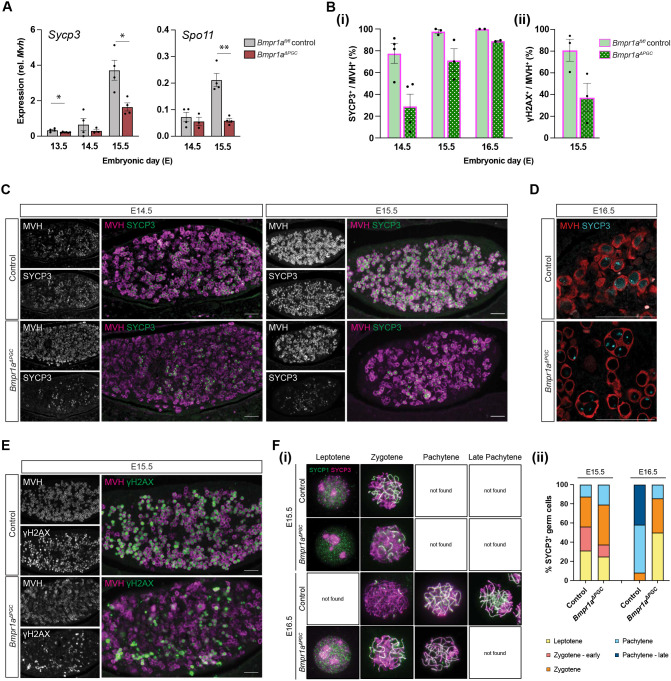
**Delayed meiotic progression in *Bmpr1a^ΔPGC^* germ cells.** (A) Expression of meiotic progression markers *Sycp3* and *Spo11* was significantly reduced in the *Bmpr1a^ΔPGC^* mutants at E13.5 and E15.5 (*Sycp3*) and E15.5 (*Spo11*). (Bi,C) Quantification of immunofluorescence revealed reduced expression of SYCP3 in mutants at E14.5-E16.5, compared to control germ cells. (Bi,D) At E16.5, most control germ cells expressed SYCP3, which decorated the length of the chromosomes; fewer mutant germ cells expressed SYCP3, which, when present, displayed a punctate nuclear pattern similar to that observed at earlier timepoints in the control. (Bii, E) At E15.5, fewer *Bmpr1a^ΔPGC^* germ cells were positive for the DNA double-stranded breaks marker γH2AX. (Fi) Immunostaining for SYCP1 (green) and SYCP3 (magenta) in meiotic chromosome spreads showed a similar distribution pattern of SYCP3^+^ cells at different stages of meiotic prophase I at E15.5 but a different distribution pattern at E16.5 between control and *Bmpr1a^ΔPGC^* cells. (Fii) At E15.5, both control and *Bmpr1a^ΔPGC^* SYCP3^+^ cells were mostly in leptotene and zygotene stages of meiotic prophase. At E16.5, >90% of SYCP3^+^ cells in the control ovaries had reached pachytene, some had reached late pachytene, and none were found in the leptotene stage. On the contrary, very few *Bmpr1a^ΔPGC^* SYCP3^+^ cells had reached pachytene, no late-pachytene cells were found, and 85% of the SYCP3^+^ cells remained in leptotene or zygotene. *P<0.05, **P<0.01 (*n*≥4, unpaired *t*-test; data are mean±s.e.m.). Cytoplasmic MVH (magenta) marks germ cells. Scale bars: 50 μm.

Despite normal onset of *Stra8*, expression of meiotic marker *Sycp3* was significantly reduced in the *Bmpr1a^ΔPGC^* mutants at E13.5 and E15.5, as was the expression of leptotene/zygotene marker *Spo11* at E15.5 (Fig. 3A). At E14.5, 77.6% of germ cells in control ovaries were SYCP3^+^, whereas only 29.1% mutant germ cells were SYCP3^+^ (Fig. 3Bi-C). By E15.5, most, if not all, control germ cells were SYCP3^+^, but the mutant ovaries still contained fewer SYCP3-expressing germ cells, consistent with the lower *Sycp3* transcription described above (Fig. 3Bi-C). By E16.5, more mutant germ cells expressed SYCP3, but the protein did not decorate the lengths of the chromosomes, as in control germ cells. Instead, we observed SYCP3 protein in a pre-leptotene-like pattern (Prieto et al., 2004) in the nucleus, as seen in controls at earlier timepoints, E14.5 and E15.5 (Fig. 3Bi,D). At E15.5, ∼80% of control but <40% of mutant germ cells were positive for the DNA double-stranded break marker γH2AX (Fig. 3Bii,E). These results suggest that germ cells in the mutant ovaries were able to initiate meiosis (express STRA8) but, in a considerable portion of them, progression into meiotic prophase, indicated by the expression of SYCP3, was delayed.

To interrogate the progression through meiotic prophase I, meiotic chromatin spreads were prepared for E15.5 and E16.5 control and mutant germ cells. Meiotic prophase sub-stages were determined by immunostaining patterns of SYCP1 and SYCP3 (Fig. 3F). At E15.5, meiotic progression was similar in control and mutant SYCP3^+^ germ cells, with most in leptotene and zygotene stages, and a small proportion reaching early pachytene. However, by E16.5, >90% of control SYCP3^+^ cells were in mid- to late-pachytene stage, while the majority of *Bmpr1a^ΔPGC^* SYCP3^+^ cells (∼85%) remained in leptotene or zygotene stages (Fig. 3F). Thus, distribution of SYCP3^+^ meiotic germ cells in various stages of prophase was similar between the control and *Bmpr1a^ΔPGC^* mutants at E15.5, but vastly different at E16.5 (Fig. 3F), suggesting a potential defect in meiotic progression in *Bmpr1a^ΔPGC^* germ cells, possibly immediately before pachytene stage.

### *Bmpr1a^ΔPGC^* germ cells abnormally retained pluripotency marker OCT4 and were slow to abandon the mitotic cell cycle

**Fig. 4.**
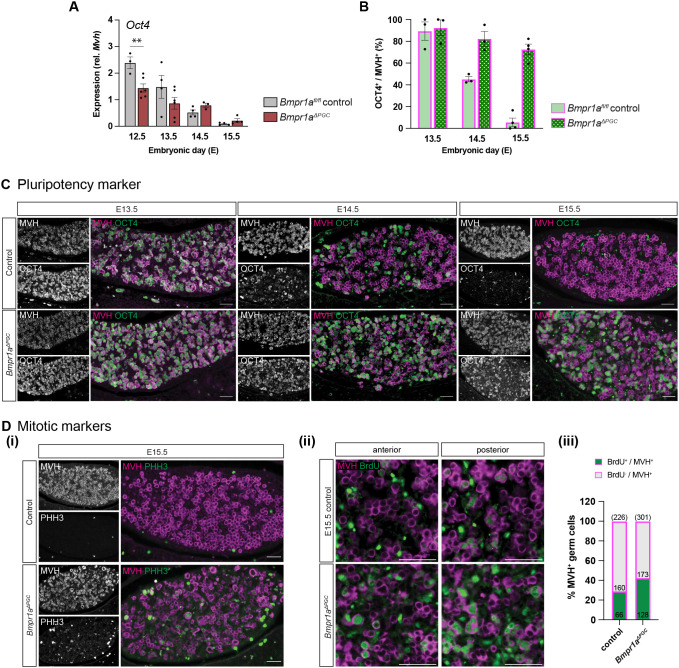
***Bmpr1a^ΔPGC^* germ cells abnormally retained OCT4 and mitotic exit was delayed.** (A) *Oct4* expression in *Bmpr1a^ΔPGC^* mutant ovaries was lower than that in the control at E12.5, but not significantly different at later timepoints. (B-Di) Control germ cells lost OCT4 gradually from E13.5 to E15.5, but *Bmpr1a^ΔPGC^* germ cells abnormally retained OCT4 expression (B,C), and maintained expression of the mitotic G_2_/M marker phospho-histone H3 at E15.5 (Di). (Dii,iii) To monitor S phase, embryos were exposed to BrdU for 2 h. Fewer germ cells (MVH^+^) in E15.5 control ovaries were positive for BrdU immunosignal compared to *Bmpr1a^ΔPGC^* germ cells. Of the cells quantified, 110/226 (48.6%, control) and 134/301 (44.5%, mutant) were located at the anterior end of the ovaries. **P<0.01 (*n*≥4, unpaired *t*-test; data are mean±s.e.m.). Cytoplasmic MVH (magenta) marks germ cells. Scale bars: 50 μm.

Entry of fetal germ cells into meiotic prophase I is marked by the loss of pluripotency-associated *Oct4*/OCT4 (Bullejos and Koopman, 2004; Menke et al., 2003; Pesce et al., 1998; Western et al., 2010). Interestingly, we found that *Oct4* expression was lower in the mutant ovaries compared to the control at E12.5, though no change was seen at later timepoints (Fig. 4A). The result was clearer when we investigated OCT4 protein. OCT4 immunostaining showed a gradual loss of the protein from E13.5 to E14.5 in the control germ cells, and an almost complete absence by E15.5, as expected (Bullejos and Koopman, 2004). In the mutants, however, OCT4 immunosignal persisted through to E15.5 with little evidence of reduction (>70% remained OCT^+^ at E15.5; Fig. 4B,C). This suggests that post-transcriptional regulation of *Oct4*/OCT4 may require BMP signalling.

To investigate the exit from mitosis, we performed immunofluorescence staining for the mitotic G2/M marker phospho-histone H3 (PHH3). At E15.5, control ovaries were completely devoid of PHH3^+^ germ cells but some germ cells in the *Bmpr1a^ΔPGC^* ovary were PHH3 positive (Fig. 4Di). Punctate mitotic prophase-like pattern and bouquet-like metaphase/telophase pattern of PHH3 (Medani et al., 2021) could be seen in some *Bmpr1a*-null germ cells, suggesting they were still proliferating mitotically. We also assessed cell cycle progression by injecting BrdU into pregnant females 2 h before embryo collection. At E15.5, BrdU signal was detected in 29.2% of MVH^+^ germ cells in the control ovaries, and 42.5% of MVH^+^ cells in the mutants (Fig. 4Dii,iii), indicating that some mutant germ cells fail to abandon the mitotic cell cycle on schedule.

### RNA-Seq analysis revealed transcriptional impacts of BMP signalling in fetal germ cells

**Fig. 5.**
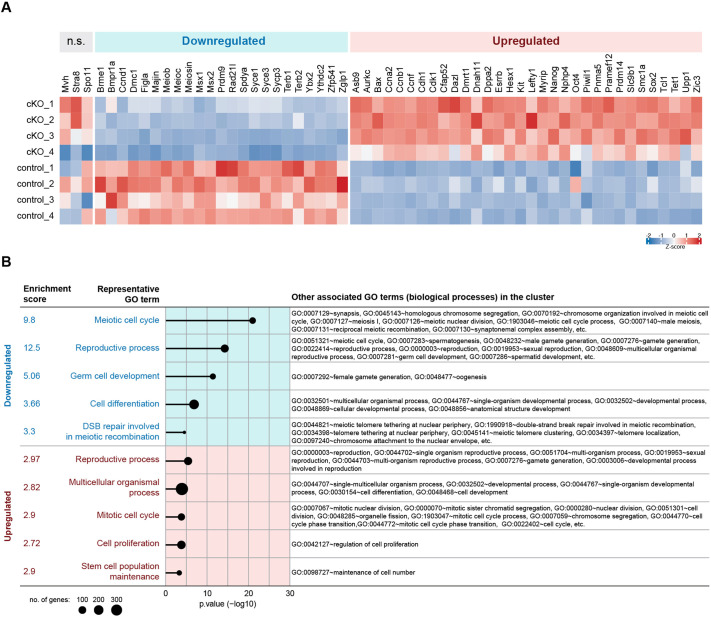
**RNA-Seq revealed aberrant gene expression in E14.5 *Bmpr1a^ΔPGC^* mutant ovaries.** RNA-Seq was conducted on E14.5 *Bmpr1a^ΔPGC^* mutant and control ovaries (*n*=4, pools of two ovary pairs each), and differentially expressed genes (DEGs) were defined as those with a |Log_2_FC|>0.5 and FDR<0.05 (downregulated, 600; upregulated, 840; see Table S1 for full list of DEGs). (A) Consistent with the *in vivo* phenotype observed, *Stra8* was not differentially expressed but some other meiosis-associated genes were downregulated, while pluripotency-associated genes were upregulated in the *Bmpr1a*-cKO. Expression levels of selected genes are visualised in the heatmap. (B) Functional annotation clustering identified an enrichment for genes associated with mitosis and stem cell population maintenance among the upregulated clusters, and an enrichment for genes associated with meiosis and germ cell development among the downregulated clusters. Full lists of enriched biological processes GO terms and the associated genes among the top 5 enriched annotation clusters are listed in Table S2 (downregulated in *Bmpr1a^ΔPGC^*) and Table S3 (upregulated in *Bmpr1a^ΔPGC^*).

Following the candidate gene/protein assessment, we then sought to evaluate the regulatory role of BMP signalling more systematically. Bulk RNA-Seq was conducted to compare the transcriptomes of E14.5 mutant and control ovaries. Differentially expressed genes (DEGs) were defined as those with |Log_2_-fold change (Log_2_FC)|>0.5 (i.e. upregulated or downregulated by at least 1.4 fold) and a false discovery rate (FDR)<0.05. Of the 14,244 genes mapped, 1440 were differentially expressed (600 downregulated and 840 upregulated) in *Bmpr1a^ΔPGC^* ovaries compared to the control (Fig. 5; Fig. S4; Table S1).

As expected, BMP signalling target *Zglp1* (Nagaoka et al., 2020) was downregulated in the *Bmpr1a*-cKO (down 2.44-fold), as were canonical BMP target genes *Msx1* (down 2.31-fold) and *Msx2* (down 3.53-fold) (Fig. 5A). Consistent with the results reported above, functional annotation of the downregulated genes found an enrichment for those associated with ‘meiotic cell cycle’, ‘reproductive process’, ‘germ cell development’, ‘cell differentiation’, and ‘double-strand break repair involved in meiotic recombination’ (Fig. 5B; Table S2). Male germ cell-related GO terms such as ‘spermatogenesis (GO:0007283)’ and ‘spermatid development (GO:0007286)’ were among the over-represented GO terms. However, we noted that many genes associated with these ‘male’ processes (e.g. *Majin, Ythdc2*, *Sycp3*, *Meioc* and *Zglp1*) are also known meiotic factors in the female germline, suggesting this enrichment is related to the meiotic process during spermatogenesis rather than to the male program per se*.*

DEGs upregulated in *Bmpr1a^ΔPGC^* ovaries were enriched for those associated with mitosis, cell proliferation, and stem cell population maintenance (Fig. 5B; Table S3), consistent with our observation that *Bmpr1a*-null germ cells exhibited a delay in early meiotic progression and the abandonment of mitosis. As observed in our candidate approach, genes that are not dependent on BMP signalling for their expression included *Mvh* and *Stra8* (Fig. 5A). Induction of *Dazl*, a marker of germ cell ‘licencing’ (Gill et al., 2011) was unaffected by the loss of *Bmpr1a*, but, rather, upregulated in the mutant (up 1.54-fold). In line with our observation of abnormal OCT4 protein retention, we found that loss of BMP signalling was associated with upregulation of pluripotency-associated genes (e.g. *Oct4*, *Sox2*, *Nanog*, *Cdh1*, *Prdm14*, *Tcl1*, *Dppa2*, *Esrrb* and *Zic3*). Moreover, some genes that are normally highly expressed by fetal testicular but not ovarian germ cells (neither pre-meiotic nor meiotic) were aberrantly upregulated in the *Bmpr1a*-cKO [e.g. *Lefty1*, *Hesx1*, *Asb9*, *Upp1* and *Pramef12* (*Pramel13*); Jameson et al., 2012; Spiller et al., 2012]. This suggests BMP signalling is required to prevent some degree of germ cell masculinisation.

### Some key genes expressed during mitosis-to-meiosis transition are BMP dependent, STRA8 dependent or co-dependent

**Fig. 6.**
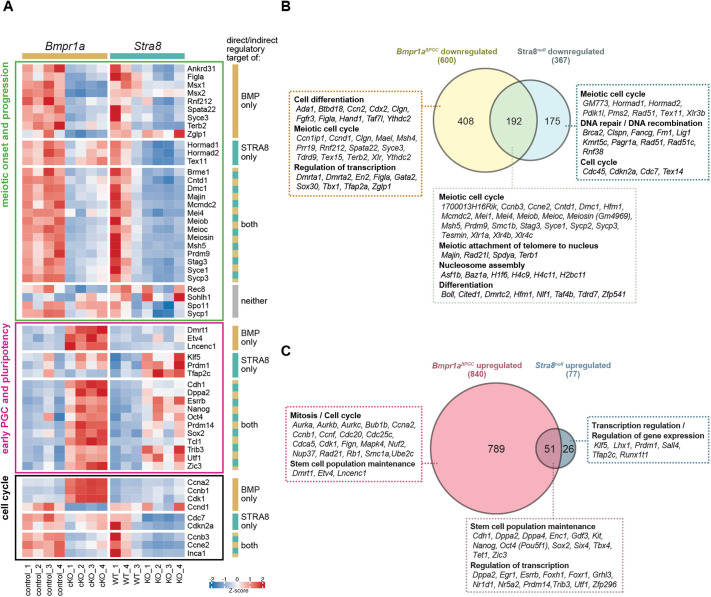
**Key genes expressed during germ cell mitosis-to-meiosis transition are BMP dependent, STRA8 dependent, or co-dependent.** DEGs in E14.5 *Bmpr1a^ΔPGC^* and *Stra8^null^* ovaries were compared to identify genes that are regulated by BMP signalling, by STRA8, or by both. (A) BMP signalling and STRA8 both independently and cooperatively regulate expression of meiotic genes, early PGC markers, pluripotency-associated genes, and cell cycle regulators. Expression of selected genes are visualised in the heatmap. (B,C) Venn diagrams represent genes that are (B) induced by BMP signalling, STRA8, or both (downregulated in mutants), or (C) repressed by BMP signalling, STRA8, or both (upregulated in mutants). See Table S4 for the full gene list.

Recent studies of PGCLCs (Miyauchi et al., 2017; Nagaoka et al., 2020) proposed that RA and BMP signalling synergistically promote oogenic germ cell fate, but the interplay between BMPs and RA/STRA8 has not been studied *in vivo*. To better understand meiotic onset in fetal ovarian germ cells, we analysed our *Bmpr1a*-cKO data alongside *Stra8*-null data (both RNA-Seq datasets derived from E14.5 whole ovaries, with DEGs defined as above). Appreciating the caveats that these single E14.5 timepoint datasets would not only identify direct downstream BMP/STRA8 targets, but would also capture differences due to delayed germ cell progression, we classified genes that were broadly (1) regulated only by BMP signalling, (2) regulated only by STRA8, and (3) regulated to some extent by both (Fig. 6; Table S4).

Comparing *Stra8^null^* versus *Stra8^WT^* returned 367 differentially downregulated and 77 differentially upregulated genes (Table S5). Interestingly, we found far fewer DEGs than the 2361 identified in a study where high-STRA8 and *Stra8*-null preleptotene cells from postnatal testis were similarly compared (Kojima et al., 2019). A large proportion of identified DEGs were also differentially expressed in the *Bmpr1a^ΔPGC^* ovaries [52.3% (192 out of 367) of downregulated genes; 66.2% (51 out of 77) of upregulated genes] (Fig. 6). Despite the considerable overlap, 408 genes were induced (downregulated in *Bmpr1a*-cKO) and 789 genes were repressed (upregulated in *Bmpr1a*-cKO) because of BMP signalling, independently of STRA8. On the other hand, we found that STRA8 induces 175 genes (downregulated in *Stra8^null^*) and represses 26 genes (upregulated in *Stra8^null^*) independently of BMP signalling. Whether these expression changes are the direct or indirect consequences of SMAD1/5/8 activation (for BMP signalling) or direct STRA8 transcriptional activity cannot be ascertained from our datasets.

#### Meiotic onset and progression genes

Our results suggest most of the known meiotic and synaptonemal complex-related genes are regulated by both BMP signalling and STRA8 (downregulated in *Bmpr1a^ΔPGC^* and *Stra8^null^* samples) (Fig. 6B; Table 1; Table S4). This group includes *Mei4*, *Cntd1*, *Stag3*, *Syce1*, and *Sycp3.* Notably, expression of *Meiosin* (*Gm4969*, encoding the interacting partner of STRA8, which is crucial for meiotic initiation; Ishiguro et al., 2020) was dependent on both STRA8 (down 6.5-fold) and BMP signalling (down 3.59-fold) (Fig. 6A; Table 1). In postnatal male germ cells, expression of *Stra8* and *Meiosin* was reported as being mutually independent, with both genes relying on RA for their expression (Ishiguro et al., 2020); thus, our results suggest a sexual dimorphic regulation of *Meiosin*, consistent with a recent report that RA does not directly regulate *Meiosin* expression in female germ cells (Shimada et al., 2023). Direct transcriptional regulation of *Meiosin* by STRA8 is plausible, as STRA8 binding sites are present on the *Meiosin* promoter region (Ishiguro et al., 2020) and expression of *Stra8* precedes that of *Meiosin* (Shimada et al., 2023). Consistently, target genes of MEIOSIN/STRA8 (e.g. *Dmc1*, *Prdm9*, *Meiob*, *Msh5*, *Mcmdc2* and *Brme1*; Ishiguro et al., 2020; Takemoto et al., 2020) were significantly downregulated in both *Bmpr1a^ΔPGC^* and *Stra8^null^* ovaries. Others found that *Meioc* upregulation at the onset of meiosis does not require RA or STRA8 (Abby et al., 2016); however, we found that *Meioc* expression is substantially affected by loss of STRA8 (down 4.07-fold) and by loss of BMP signalling (down 3.31-fold). This is in line with evidence that *Meioc* expression is dependent on STRA8 (Soh et al., 2015), and that its promoter region is bound by MEIOSIN and STRA8 (Ishiguro et al., 2020).

**Table 1.** Expression of meiotic genes in *Bmpr1a^ΔPGC^* and *Stra8^null^* mutants

Gene	Function in the context of meiosis	Female KO reproductive phenotype	*Stra8* null*	*Bmpr1a* cKO*	Reference
*Ankrd31*	Double-stranded break (DSB) formation	Premature sterility	n.s.	0.37	Papanikos et al. (2019)
*Cntd1*	Homologous recombination	Sterile	0.32	0.59	Holloway et al. (2014)
*Dmc1*	DSB repair	Sterile	0.19	0.47	Pittman et al. (1998); Yoshida et al. (1998)
*Hfm1*	Crossover formation and complete synapsis	Sterile	0.32	0.31	Guiraldelli et al. (2013)
*Figla*	Transcription factor for oocyte-specific gene expression	Lack of ovarian follicle formation	0.26^‡^	0.24	Soyal et al. (2000)
*Hormad1*	Promotes homolog alignment and synaptonemal complex (SC) formation	Sterile	0.44	n.s.	Shin et al. (2010)
*Hormad2*	Removal of asynaptic oocytes	Fertile	0.40	0.74	Kogo et al. (2012); Wojtasz et al. (2012)
*Iho1* (*Ccdc36*)	DSB formation	Sterile	0.12	0.33	Stanzione et al. (2016)
*Kash5* (*Ccdc155*)	Chromosome synapsis	Sterile	0.18	0.35	Horn et al. (2013)
*M1ap*	Crossover formation	Fertile	0.16	0.34	Li et al. (2023)
*Majin*	Meiotic telomere complex protein	Sterile	0.06	0.38	Shibuya et al. (2015)
*Mcmdc2*	Meiotic recombination	Sterile	0.27	0.38	Finsterbusch et al. (2016)
*Mei1*	Chromosome synapsis	Sterile	0.04	0.13	Libby et al. (2003)
*Mei4*	DSB formation	Nearly devoid of primordial and primary follicles	0.32	0.58	Kumar et al. (2010)
*Meiob*	Meiotic recombination	Sterile	0.16	0.17	Souquet et al. (2013)
*Meioc (Gm1564)*	Post-transcriptional regulator	Sterile	0.25	0.30	Abby et al. (2016); Soh et al. (2015, 2017)
*Meiosin* (*Gm4969*)	STRA8 co-factor	Sterile	0.15	0.28	Ishiguro et al. (2020)
*Mlh1*	DNA mismatch repair and crossing over	Sterile	n.s.	n.s.	Baker et al. (1996)
*Msh4*	Chromosome pairing	Sterile	0.48^§^	0.48	Kneitz et al. (2000)
*Msh5*	DSB repair	Sterile	0.23	0.43	de Vries et al. (1999)
*Prdm9*	Epigenetic events in meiotic prophase; determines sites of DSB	Sterile	0.07	0.23	Baudat et al. (2010); Hayashi et al. (2005)
*Rad21l*	Cohesin to link homologous chromosomes	Pre-mature sterility	0.05	0.18	Herrán et al. (2011); Ishiguro et al. (2011); Lee and Hirano (2011)
*Rec8*	Meiotic-specific cohesin	Sterile	n.s.	0.71	Bannister et al. (2004); Xu et al. (2005)
*Rnf212*	Homologous recombination	Sterile	n.s.	0.36	Reynolds et al. (2013)
*Slc25a31* (*Ant4*)	Adenine nucleotide translocase	Fertile with smaller litter size	0.40	n.s.	Lim et al. (2015)
*Smc1b*	Meiotic-specific cohesin	Sterile	0.33	0.48	Revenkova et al. (2004)
*Sohlh1*	Oocyte differentiation-related transcription factor	Sterile	n.s.	n.s	Pangas et al. (2006); Shin et al. (2017)
*Spata22*	Meiotic prophase progression	Sterile	0.16^¶^	0.20	La Salle et al. (2012)
*Spo11*	Catalyse DSBs	Sterile (premature ovarian failure)	n.s.	n.s.	Baudat et al. (2000); Keeney et al. (1997)
*Stag3*	Meiotic-specific cohesin	Sterile	0.39	0.57	Hopkins et al. (2014)
*Stra8*	Meiosis-specific transcription factor	Sterile	KO	n.s.	Baltus et al. (2006)
*Sun1*	Homologous pairing and synapsis formation	Sterile	n.s.	n.s.	Ding et al. (2007)
*Syce1*	SC structural protein	Sterile	0.30	0.55	Bolcun-Filas et al. (2009)
*Syce3*	SC structural protein	Sterile	n.s.	0.32	Schramm et al. (2011)
*Sycp1*	SC structural protein	Sterile	n.s.	0.72	de Vries et al. (2005)
*Sycp2*	SC structural protein	Subfertile	0.24	0.49	Yang et al. (2006)
*Sycp3*	SC structural protein	Subfertile	0.26	0.39	Yuan et al. (2002)
*Taf4b*	Component of the Transcription factor IID (TFIID) complex	Sterile	0.52	0.69	Grive et al. (2014)
*Taf7l*	Component of the TFIID complex	Fertile	0.33^§§^	0.49	Cheng et al. (2007)
*Terb1*	Meiotic telomere complex protein	Sterile	0.09	0.26	Shibuya et al. (2014)
*Terb2*	Meiotic telomere complex protein	Sterile	0.27**	0.48	Shibuya et al. (2015)
*Tex11*	DSB repair and regulation of crossing over	Subfertile	0.30	n.s.	Yang et al. (2008)
*Tex12*	SC structural protein	Sterile	0.20	0.39	Hamer et al. (2008)
*Top6bl* (*Gm960*)	DSB formation; forms a complex with SPO11	Few primordial and primary follicles	0.17	0.49	Robert et al. (2016)
*Ythdc2*	Post-transcriptional regulator	Sterile	0.68^‡‡^	0.71	Bailey et al. (2017)
*Zglp1*	Oogenic program activator	Sterile	n.s.	0.41	Nagaoka et al. (2020)

*Expression levels relative to the control.

^‡^*P*=0.064, ^§^*P*=0.060, ^¶^*P*=0.051, **P=0.063, ^‡‡^*P*=0.068, ^§§^*P*=0.1447.

n.s., not significant.

Although a large subset of meiotic genes is regulated by both BMP signalling and STRA8, some genes are independently regulated by either BMP or STRA8 (Fig. 6; Table 1; Table S4). Genes downregulated only in *Bmpr1a^ΔPGC^* ovaries include *Zglp1*, *Figla*, *Msx1*, *Msx2*, *Syce3*, *Terb2*, *Ankrd31*, *Rnf212*, and *Spata22*. Genes that are expressed independently of BMP signalling but are STRA8 dependent include *Hormad1*, *Hormad2*, and *Tex11*.

Interestingly, some genes known to be upregulated at meiotic onset were expressed normally in the absence of either BMP signalling or STRA8 protein, suggesting their induction might depend on RA, RA-responsive factors other than STRA8, unknown signalling factors present in the fetal gonad, or synergistic actions of BMP signalling and STRA8 (Table 1). As previously reported, *Rec8* expression was fully independent of STRA8 (Soh et al., 2015), and its expression was reduced only marginally in the *Bmpr1a-*cKO (down 1.41-fold). This group also includes *Sycp1*, *Spo11*, and *Sohlh1*.

#### Markers of early PGCs and pluripotency

Diminished expression of early PGC markers and pluripotency-related genes upon meiotic entry could be regulated by STRA8, BMP signalling, or both (Fig. 6C; Table S4). Those with expression apparently repressed by STRA8 (upregulated in *Stra8^null^*) but not BMP signalling include *Tfap2c*, *Prdm1*, and *Klf5*, whilst downregulation of *Dmrt1*, *Etv4*, and *Lncenc1* was dependent on BMP signalling but not STRA8. Genes that appear to be downregulated by both (upregulated in both *Stra8^null^* and *Bmpr1a^ΔPGC^* ovaries) include *Dppa2*, *Nanog*, *Oct4*, *Sox2*, *Prdm14*, *Trib3,* and *Utf1*.

#### Genes potentially associated with the mitosis-to-meiosis switch

Consistent with findings that cell cycle genes are dynamically regulated during meiosis initiation (Zhao et al., 2020), such genes were aberrantly expressed in both *Bmpr1a^ΔPGC^* and *Stra8^null^* ovaries (Fig. 6B,C; Table S4). We show that BMP signalling represses expression of *Ccnb1* and *Ccna2* but seems responsible for upregulating *Ythdc2*; elevation of YTHDC2 is necessary for downregulating *Ccna2* during the mitotic-to-meiotic transition (Bailey et al., 2017). Elevated expression of *Ccnd1* at meiotic onset also relies on intact BMP signalling. Although it is generally accepted that cyclin-dependent kinases are constitutively present during the cell cycle while levels of cyclins fluctuate cyclically, we find evidence that BMP signalling downregulates *Cdk1* expression*.* This could be functionally relevant, as decreased expression of Cdk genes is also observed in postnatal testicular germ cells as they progress into meiosis (Diederichs et al., 2005). In agreement with findings from a recent study (Shimada et al., 2023), our data indicate that STRA8 is required to upregulate *Cdkn2a*, and further clarifies that it is only the p19^ARF^-encoding isoform of *Cdkn2a* that is upregulated when STRA8 is present.

Our data support the theory that BMP signalling and STRA8 act together to influence the mitosis-to-meiosis switch. *Ccne2*, which is upregulated in early meiotic germ cells and encodes a cyclin required in meiotic prophase I, at least in males (Martinerie et al., 2014), was significantly downregulated in both *Stra8^null^* and *Bmpr1a^ΔPGC^* E14.5 ovaries. Expression of the meiosis-specific *Ccnb3* also requires both STRA8 and BMP signalling. Another gene regulated by both BMP signalling and STRA8 is *Inca1*, encoding a novel Cyclin A1/CDK2 inhibitor expressed in male and female germ cells as they enter meiotic prophase (Diederichs et al., 2005; Jameson et al., 2012). Overall, our results support a multi-faceted role for BMP signalling in instructing germ cell meiosis in female mice *in vivo*, and demonstrate the importance of both BMP signalling and STRA8 in ensuring a proper mitosis-to-meiosis transition.

## DISCUSSION

**Fig. 7.**
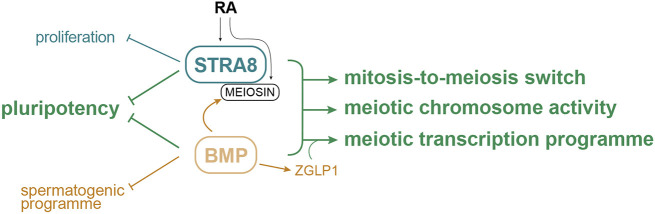
**Both BMP signalling and STRA8 are required for fetal ovarian germ cell meiosis *in vivo*.** This article demonstrates an essential role for BMP signalling in fetal germ cell meiosis. We conclude that BMP signalling and STRA8 both directly or indirectly contribute to downregulating pluripotency in differentiating germ cells, and cooperatively ensure a proper mitotic-to-meiotic switch and activate factors required for meiotic chromosome activity. Through ZGLP1, BMP signalling in germ cells also promotes a meiotic transcription program. BMP signalling and STRA8 also independently promote germ cell meiosis by downregulating the spermatogenic program and repressing proliferation, respectively.

Successful mitosis-to-meiosis transition is essential for gametogenesis. In mammalian females, this process occurs during embryogenesis, giving rise to the non-renewable ovarian reserve. Evidence indicates that ovarian germ cells respond to endogenous RA by expressing the transcription factor STRA8, which drives meiotic initiation (Bowles et al., 2006; Feng et al., 2021; Koubova et al., 2006; Soh et al., 2015). Recent findings from mouse PGCLC studies suggest that BMP signalling is also required, at least *in vitro*, for adoption of the oogenic fate (Miyauchi et al., 2017). Such *in vitro* studies are powerful and allow a relatively simple analysis of germ cell sex determination ‘in a reductive and a constructive fashion’ (Miyauchi et al., 2017). Nonetheless, it remains uncertain as to whether and how BMP signalling impacts ovarian germ cell development *in vivo*. In this study, we provide *in vivo* evidence that expression of *Stra8* is independent of BMP signalling but expression of *Meiosin*, which encodes the STRA8-interacting protein, is partially dependent on BMP signalling, in the mouse fetal ovary. Combining transcriptome analyses of *Bmpr1a^ΔPGC^* and *Stra8^null^* fetal ovaries, we demonstrate that BMP signalling and STRA8 have independent but also common targets for ensuring proper expression of meiotic factors and downregulation of pluripotency (Fig. 7).

BMP signalling is important in many aspects of fetal germ cell development; here, we aimed to investigate the function of BMPs in post-migratory germ cells with a view to confirming a role in sexual fate commitment. Noting that BMP2 and BMP5 are likely to be present in the fetal ovary, but not the fetal testis (Jameson et al., 2012; Yao et al., 2004), we chose to genetically delete *Bmpr1a*, encoding the BMP receptor BMPR1A, specifically in the germ cells after gonadal colonisation. Both BMP2 and BMP5 signal through BMPR1A and BMPR1B (ALK6), and BMP5 additionally uses ACVR1 (Mueller and Nickel, 2012). *Bmpr1a* expression is high, and *Bmpr1b* expression is negligible in fetal germ cells, with low expression detected for *Acvr1* (Jameson et al., 2012). Whilst we cannot exclude the possibility that BMPs could still signal through ACVR1 in our *Bmpr1a*-deleted model, we expect that BMPR1A-mediated signalling from soma to germ cells is largely abrogated. Our near-complete loss of BMPR1A immunostaining along with the BMP signalling effector *Zglp1* confirmed that the model was appropriate for our *in vivo* investigation.

### BMP signalling is not required for initiation of *Stra8* expression but is essential for meiotic entry and progression

The requirement for STRA8 in female germ cell meiosis is unequivocal (Baltus et al., 2006); however, the necessity for RA in this context has been a long-standing debate (Chassot et al., 2020; Kumar et al., 2011; Vernet et al., 2020). Although we acknowledge the controversy, here we are not focusing on the question of whether RA is required to induce *Stra8* expression. Rather, we set out to determine whether BMP signalling is crucial, alongside STRA8, to ensure correct transition from mitosis to meiosis *in vivo*.

Using our germ cell-specific *Bmpr1a*-cKO model, we showed that the loss of BMP signalling does not abrogate the timely onset, level of expression, or the anterior-to-posterior induction pattern of *Stra8* in fetal ovaries. These results support the hypothesis that *Stra8* induction is driven by RA, as this signalling factor is present at higher levels at the anterior end of the ovary (Bowles et al., 2006). It is, of course, formally possible that non-RA and non-BMP signalling factors, which are present particularly at the anterior end of the gonad, are involved. This result is consistent with findings in PGCLCs, where provision of RA alone was just as successful in inducing full activation of *Stra8* expression as was RA together with BMP2 (Miyauchi et al., 2017).

Despite normal onset of *Stra8* expression, its downregulation is perturbed, and there is elevated cytoplasmic accumulation of STRA8 protein in *Bmpr1a*-null germ cells after E14.5. It is possible that elevated *Stra8* expression results in increased STRA8 protein production; if this was to saturate its active nuclear import (Tedesco et al., 2009), then STRA8 might accumulate in the cytoplasm. Another more-specific mechanism could relate to the reduced expression of *Meiosin* in germ cells in the absence of BMP signalling (Fig. 5A). In mouse spermatogenic cells, loss of *Meiosin* led to cytoplasmic accumulation of STRA8 (Ishiguro et al., 2020), a phenotype similar to that observed in our mutant ovaries. Whether MEIOSIN regulates the nuclear import of STRA8 in fetal ovaries requires further investigation. With respect to aberrant maintenance of *Stra8* expression in *Bmpr1a*-null germ cells, increased expression of *Dmrt1* (Fig. 5A) could enhance and possibly maintain expression of *Stra8* through the binding of DMRT1 to its proximal promoter (Feng et al., 2021; Krentz et al., 2011). More generally, it is possible that *Stra8* expression is maintained in the absence of BMP signalling because meiosis is not progressing or, more specifically, because there is insufficient nuclear STRA8 to downregulate its own expression (Soh et al., 2015). Future assessment of the expression, (co)localisation, and modification of STRA8 and MEIOSIN would help shed light on these possibilities.

Although *Stra8* expression is initiated normally, we find that loss of BMP signalling causes a delay in meiotic prophase entry and progression. In *Bmpr1a^ΔPGC^* ovaries, transcription of key meiotic genes was significantly reduced, fewer germ cells were positive for SYCP3, and those that did express SYCP3 were temporally delayed in meiotic progression. However, despite very low *Zglp1* expression, our phenotype did not recapitulate the near complete absence of SYCP3 observed in the ubiquitous *Zglp1^−/−^* model (Nagaoka et al., 2020), possibly due to the specificity of our knockout to post migratory germ cells: in the *Zglp1*^−/−^ model, ZGLP1 would have been absent throughout development in germ cells and somatic cells. We were unable to analyse postnatal phenotypes because treatment with 4-hydroxytamoxifen precludes parturition (Savery et al., 2020). Future studies with non-inducible CRE lines that are active in early gonadal germ cells (Burnet et al., 2023) will be required to assess the adult phenotype.

### Both BMP signalling and STRA8 are required for proper mitosis-to-meiosis transition *in vivo*

Transcriptome profiling of *Bmpr1a^ΔPGC^* ovaries allowed identification of direct or indirect targets of BMP signalling in fetal ovarian germ cells. Comparison of *Bmpr1a*-cKO and *Stra8^null^* datasets further confirmed that both factors are indispensable for fetal germ cell meiosis. Regulatory targets of STRA8 have been reported for males (Kojima et al., 2019) but not yet for females. Further studies to identify and verify factors immediately downstream of BMP signalling and STRA8 in the context of germ cell mitosis-to-meiosis transition would be valuable for elucidating this complicated mechanism.

Our combined transcriptome analyses revealed that a plethora of cell cycle genes are independent or shared targets of BMP signalling and/or STRA8. For example, we find that expression of *Ccnd1* is dependent on BMP signalling. Despite Cyclin D1 being considered a proliferation-associated G1 mitotic marker, *Ccnd1* transcript expression increases sharply in early meiotic germ cells, and is downregulated during later stages of meiosis in an anterior-to-posterior wave (Heaney et al., 2012; Zhao et al., 2020). Thus, Cyclin D1 potentially has a role in the mitosis-to-meiosis transition and its expression is induced by BMP. We find that STRA8 is required for *Cdkn2a* transcription, as has recently been highlighted (Shimada et al., 2023). *Cdkn2a* encodes alternative isoforms p16^INK4a^ and p19^ARF^, both of which can reduce CDK activity, thereby directly (p16^INK4a^) or indirectly (p19^ARF^) impacting cell cycle progression (Chin et al., 1998). We extend what is known regarding these cell cycle regulators as we find that STRA8 upregulates only the isoform encoding p19^ARF^, and that BMP signalling is not required for *Cdkn2a* induction. In the mouse testis, the p19^ARF^ variant is transiently expressed in spermatogonia (Gromley et al., 2009) and prevents spermatocytes from undergoing p53-dependent apoptosis, thereby supporting meiotic progression (Churchman et al., 2011). In the females, however, loss of *Arf* caused no discernible reproductive defects (Churchman et al., 2011). The functional importance of p19^ARF^ expression at the mitosis-to-meiosis transition in ovarian germ cells remains uncertain and warrants further investigation.

### BMP signalling likely also affects germ cell development prior to sexual fate determination

Whilst our findings on the role of BMP signalling in female germ cell fate specification are mostly in line with the observations from PGCLCs (Miyauchi et al., 2017; Nagaoka et al., 2020), we noted a few discrepancies between the behaviour of gonadal germ cells and PGCLCs*.* In our *Bmpr1a*-cKO model, loss of BMP signalling did not affect the expression of germ cell marker *Mvh* and ‘licensing factor’ *Dazl*; this is in contrast to the report from Miyauchi et al. (2017), where inhibition of the BMP signalling pathway by LDN193189 injection (to pregnant dams) impaired *Mvh* and *Dazl* expression in fetal ovarian germ cells. Since LDN193189 treatment inhibits BMP signalling in both germ cell and gonadal somatic cells, and, further, because induction of MVH in germ cells seems to require an intercellular interaction with gonadal somatic cells (Toyooka et al., 2000), these disparate results raise the interesting possibility that normal *Mvh* induction in ovarian germ cells requires BMP signalling, but not direct signalling to germ cells. Moreover, LDN193189 treatment in pregnant dams resulted in reduced *Mvh* expression in XX but not XY germ cells (Miyauchi et al., 2017): it is possible that upregulation of *Mvh*, upon gonadal colonisation (Seligman and Page, 1998; Toyooka et al., 2000), is driven by different mechanisms in fetal ovary and fetal testis, and that BMP plays a role in driving ovarian somatic cell fate rather than directly acting on germ cells in this context. As PGCLCs transcriptionally resemble migratory PGCs rather than gonadal PGCs (Ohta et al., 2017), it seems likely that one function of BMP in the *in vitro* system is to mature PGCLCs to a state that is responsive to fate-determining signals provided by the gonadal environment.

### Conclusion

A role for BMPR1A-mediated signalling in ensuring correct fetal ovarian germ cell meiosis is now unequivocal. Our *in vivo* data support most of the conclusions reached by Miyauchi et al. (2017) and Nagaoka et al. (2020) in their studies of PGCLCs. Importantly, our findings support the contention that the PGCLC system recapitulates, to a great extent, germ cell development *in vivo.* Our work helps clarify the *in vivo* mechanisms that underlie commitment of germ cells to the female fate – information that may help in the quest to increase efficiency and derive safe and effectives oocytes, *in vitro*, for eventual therapeutic use. Our data also contribute to the knowledgebase with respect to the question of how germ cells lose pluripotency and differentiate – information that is relevant to stem cell biology more broadly.

## MATERIALS AND METHODS

### Animals

All procedures involving animals and their care were carried out in accordance with Australian national, state, and institutional guidelines. Breeding of mice and animal experiments were approved by The University of Queensland Animal Ethics Committee. The *Bmpr1a^tm2.1Bhr^* (referred to as *Bmpr1a^fl/fl^* in this work), *Oct4-MerCreMer* (referred to as *Oct4-CreERT2* in this work) and *Stra8^Δ173^* (referred to as *Stra8^null^* in this work) mouse lines have been described by Mishina et al. (2002), Greder et al. (2012), and Feng et al. (2021), respectively. All mouse lines were maintained on a pure C57BL/6 background; imported *Bmpr1a^fl/fl^* animals were received on a C57BL/6×129SV background, and subsequently backcrossed with wild-type C57BL/6 for more than nine generations to obtain a largely C57BL/6 background. *Oct4-Cre^ERT2^* animals were intercrossed with *Bmpr1a^fl/fl^* animals to produce *Bmpr1a^fl/fl^;Oct4-Cre^Cre/WT^* male studs for timed matings with *Bmpr1a^fl/fl^* females*.* All mice were housed at the University of Queensland Biological Resources, Queensland Bioscience Precinct, Research Animal Facility with a 12 h light/dark cycle. Genotyping of the mice were performed by PCR as described previously (Chuma and Nakatsuji, 2001; Greder et al., 2012). Primer sequences are listed in Table S6.

### Timed matings and tissue collection

For embryo collections, timed matings were set up and noon of the day on which a vaginal plug was observed was designated as 0.5 days post coitum (dpc)/embryonic day (E) 0.5. Wild-type C57BL/6 males and females were housed together for timed matings for *ex vivo* gonad cultures. Embryos were collected at E11.5, and urogenital ridges (UGRs; gonad plus adjacent mesonephros) were dissected out for *ex vivo* gonad culture. For *in vivo* experiments, *Bmpr1a^fl/fl^;Oct4-Cre^Cre/WT^* studs and *Bmpr1a^fl/fl^* females were time-mated. Pregnant females were injected intraperitoneally with 1 mg 4-hydroxytamoxifen (4-OHT) (H6278, Sigma-Aldrich) and 0.5 mg progesterone (P0130, Sigma-Aldrich) at 9.5, 10.5 and 11.5 dpc to induce germ cell-specific CRE-dependent recombination of *Bmpr1a* in the embryos. Pregnant females were euthanised, and *Bmpr1a^fl/fl^;Oct4-Cre^Cre/WT^* (referred to as *Bmpr1a^ΔPGC^* or *Bmpr1a*-cKO in this article) embryos and *Bmpr1a^fl/fl^* control littermates were collected (at timepoints ranging from E12.5 to E16.5) for subsequent analysis.

Embryo sex was determined by visual inspection of dissected gonads (for embryos aged E12.5 or older) while *Ube1* genotyping using tail tissue (Chuma and Nakatsuji, 2001) was performed to confirm the sex of samples collected before E12.5 or for whole embryo collections (all ages). For gene expression and immunofluorescence analyses, E12.5 – E15.5 right gonads were dissected with the mesonephroi removed and stored in RNA*later* (R0901, Merck) at 4°C until RNA extraction. Left gonads were left in the embryos and fixed in 4% paraformaldehyde in PBS (PFA/PBS), dehydrated through an ethanol series (25%, 50% and 75% v/v in water), processed (Leica ASP300 S Tissue Processor), and embedded in paraffin for sectioning and histological staining.

### Organ culture

Freshly dissected C57BL/6 E11.5 UGRs were cultured in 24-well plates in hanging drops (McClelland and Bowles, 2016) of 45 μl StemPro-34 SFM (10639011, Gibco) for 24, 48, or 72 h at 37°C in 5% CO_2_, replacing with fresh medium (including treatments where relevant) every 24 h. UGRs were treated with LDN193189 (500 nM in DMSO, SML0559, Sigma-Aldrich) or DMSO (D2650, Sigma-Aldrich). For each embryo, one UGR was used in the control group and one in the treatment group. Cultured samples were collected in pools of 2-4 and stored in RNA*later* (AM7021, Thermo Fisher Scientific) at 4°C until RNA extraction.

### Nucleic acid extraction

Genomic DNA samples for genotyping was extracted from ear notches, toe clippings, or embryonic tail using QuickExtract Solution (QE0905, Lucigen) according to the manufacturer's protocol. Total RNA was extracted from cultured UGRs, individual gonads, or gonad pairs using the RNeasy Micro Kit (74004, Qiagen), including on-column DNase treatment, according to the manufacturer's protocol.

### Quantitative reverse transcription PCR (qRT-PCR)

Reverse transcription was performed immediately after RNA extraction to synthesise cDNA using the High-Capacity cDNA Reverse Transcription Kit (4368813, Applied Biosystems). TaqMan Gene Expression Assays (Table S7) and Universal TaqMan Master Mix (4318157, Applied Biosystems) were used for gene expression quantification. Realtime-PCR runs were performed on a QuantStudio 7 Flex Real-Time PCR System (Applied Biosystems). Expression of germ cell-specific transcripts was normalised against the expression level of *mouse vasa homolog* (*Mvh*, also known as *Ddx4*), a marker of germ cells, to control for germ cell number. Expression levels of other genes were normalised against the house-keeping gene *Tbp.*

### Histological sectioning and staining

Whole embryos with right gonad excised (retained for RNA extraction) were collected for histological analyses and fixed in 4% paraformaldehyde in PBS (PFA/PBS), dehydrated in ethanol series (25%, 50%, 75% and 100% v/v in water), and embedded in paraffin wax for sectioning. Whole embryos were serial-sectioned at 5 µm on a Leica RM Rotary Microtome, and dewaxed in xylene, followed by rehydration through an ethanol series from 100% to 35% v/v in water. Immunofluorescence was performed as previously described (Bowles et al., 2010). Primary and secondary antibodies used are listed in Table S8. Images were obtained with a DP70 colour camera (Olympus) on a BX-51 upright florescence microscope (Olympus), or a LSM900 Fast AiryScan2 Confocal microscope (Zeiss). All immunofluorescence images are representative images of *n*≥3; analyses were conducted in FIJI on single images. For measuring cell roundness, cell boundaries were manually defined based on MVH immunosignal, ‘Roundness’ was measured using the ‘Measure’ function. For quantification of STRA8^+^, SYCP3^+^, OCT4^+^, or BrdU^+^ germ cells, the ‘Cell Counter’ plug-in was used for cell counting from at least two serial sections (every tenth) of each biological replicate, except for BrdU, which was counted from image sections from anterior or posterior ends. For STRA8 subcellular localisation, cytoplasmic and nuclear signal max. intensity was compared using the ‘Plot Profile’ function.

### Meiotic chromatin spreads

Meiotic chromatin spreads were prepared from E15.5 and E16.5 ovaries as per Hwang et al. (2018), with the following modifications. Briefly, ovaries were placed in 0.5 ml freshly made Hypotonic Extraction Buffer [HEB (pH 8.2-8.4); 30 mM Tris-HCl (pH 7.2), 50 mM sucrose, 17 mM trisodium citrate dihydrate, 5 mM EDTA, 0.5 mM DTT and 0.1 mM PMSF] in a 12-well plate and incubated on ice for 15-30 min. After incubation, each ovary was placed in 25 µl of 100 mM sucrose on a microscopic slide, within a square pre-drawn using hydrophobic barrier PAP pen (00-8877, Invitrogen). Cells from the ovaries were released by teasing open the ovary using 27 G needles, and carefully dispersed by pipetting. Cells were fixed in the squares with 40 µl 1% paraformaldehyde/0.15% Triton X (pH 9.2), and the slides were incubated overnight in a humid chamber at room temperature. Slides were air-dried on day 2 and stored at −80°C until immunofluorescence staining.

Immunofluorescence staining reagents were prepared as follows: antibody dilution buffer (ADB) – 3 g BSA (BSAS 0.05, Bovogen), 10 ml horse serum, 250 µl 20% Triton X and 10 ml 10×PBS made up to 100 ml with water; ADB/PBS solution – 10% ADB solution in PBS; PBTX solution – 0.1% Triton X in PBS; Photoflo/PBS – 0.4% Kodak Photo-Flo 200 Solution in PBS; Photoflo/H_2_O – 0.4% Kodak Photo-Flo 200 Solution in water; anti-fade mounting media – 0.932 g DABCO, 3.2 ml water, 800 µl 1 M Tris-HCl (pH 8.0), 36 ml glycerol and 40 µl DAPI (0.2 mg/ml).

Chromatin spreads slides were blocked for 10 min each in Photoflo/PBS, PBTX, and ADB/PBS, and incubated overnight at room temperature with primary antibodies: rabbit polyclonal anti-SYCP1 (ab15090, Abcam) and mouse monoclonal anti-SYCP3 (ab97672, Abcam) diluted at 1:200 in ADB. The blocking steps were repeated prior to incubation with secondary antibodies at 37°C for 1 h. Secondary antibodies used were: goat anti-rabbit IgG (H+L) Alexa Fluor 488 (A11034, Invitrogen) and goat anti-mouse IgG (H+L) Alexa Fluor 594 (A11032, Invitrogen), both of them at a concentration of 1:2000 diluted in ADB. Slides were washed three times in Photoflo/PBS, once in Photoflo/H_2_O, and then air-dried in the dark before mounting with 60 µl antifade mounting media. Chromatin spreads were visualised and imaged at 100× with DP70 colour camera (Olympus) on a BX-51 upright florescence microscope (Olympus). Meiotic prophase I substages for SYCP3^+^ cells were classified based on the synaptonemal complex structure visualised by anti-SYCP1 and anti-SYCP3 as follows: leptotene – synaptonemal complex starts to accumulate on chromosome, fragmented SYCP1 and SYCP3 signals; zygotene – chromosomes show signs of condensation, axes of the chromosomes marked by long thread-like SYCP3 signals with SYCP1 signals colocalised in some regions undergoing synapsis; pachytene – chromatin fully condensed and synapsed, SYCP1 and SYCP3 signals colocalised and appearing shorter and thicker than previous stages; late pachytene – chromatin shows early signs of SYCP1 dissociation from the lateral elements.

### Magnetic-activated cell sorting

Gonads collected at E13.5, were dissociated using Cell Dissociation Buffer (13151014, Gibco) for 15 min at 37°C followed by pipetting up and down until a single cell suspension was formed. Germ cells were enriched from the cell suspension using anti-SSEA-1 (CD15) microbeads (130-094-530, Miltenyi Biotec) on MS columns as per the manufacturer's protocol.

### Bulk RNA sequencing and data analysis

Fetal ovaries were collected from eight E14.5 *Bmpr1a^ΔPGC^* embryos and eight *Bmpr1a^fl/fl^* littermate controls across six litters; gonad pairs were pooled into eight samples (*n*=4 each of mutant and control, two ovary pairs per sample) and total RNA was freshly isolated as described above. cDNA libraries were constructed from 500 ng total RNA using NEBNext Ultra II Directional RNA Library Prep Kit for Illumina (E7760S, NEB), optimised for an approximate final library size of 520 bp, following manufacturer's protocols. Strand-specific paired-end libraries were sequenced on Illumina NovaSeq with a read length of 150 bp at the Australian Genome Research Facility (AGRF), Melbourne.

Reads were cleaned using Trimmomatic (Bolger et al., 2014) and mapped to the *Mus musculus* genome (mm10) using STAR 2.5.2 (Dobin et al., 2013), and read counts per gene were quantified using the same tool. Differential gene expression analysis was conducted using edgeR (Robinson et al., 2010) with the GLM approach.

Differential gene expression data were visualised using R, sashimi plots were generated with the tool *ggsashimi* (Garrido-Martín et al., 2018), and Gene ontology (GO) analysis was performed using DAVID (Huang et al., 2009; Sherman et al., 2022), using all expressed genes in the samples as the background.

### Statistical analyses

Statistical analyses were conducted using Prism 9, *n*≥3 unless otherwise stated, graphs were plotted as mean±s.e.m. for TaqMan gene expression analyses and protein localisation analyses, and as mean±s.d. for cell roundness. Paired *t*-test (two-tailed) was used to calculate statistical significance in *ex vivo* gonad culture experiments; unpaired *t*-test (two-tailed) was used to calculate statistical significance in *in vivo* experiments.

## Supplementary Material

10.1242/develop.204227_sup1 Supplementary information

Table S1. Differentially expressed genes between E14.5 *Bmpr1a*-cKO and *Bmpr1a*-flox control ovaries.

Table S2. Enriched biological processes GO terms associated with the top 5 annotated clusters for differentially downregulated genes in *Bmpr1a*-cKO.

Table S3. Enriched biological processes GO terms associated with the top 5 annotated clusters for differentially upregulated genes in *Bmpr1a*-cKO.

Table S4. Differentially expressed genes in E14.5 *Bmpr1a*-cKO and/or *Stra*8-null ovaries.

Table S5. Differential gene expression result for E14.5 *Stra*8-null vs WT control ovaries.

## References

[DEV204227C1] Abby, E., Tourpin, S., Ribeiro, J., Daniel, K., Messiaen, S., Moison, D., Guerquin, J., Gaillard, J.-C., Armengaud, J., Langa, F. et al. (2016). Implementation of meiosis prophase I programme requires a conserved retinoid-independent stabilizer of meiotic transcripts. *Nat. Commun.* 7, 10324. 10.1038/ncomms10324 26742488 PMC4729902

[DEV204227C2] Bailey, A. S., Batista, P. J., Gold, R. S., Chen, Y. G., De Rooij, D. G., Chang, H. Y. and Fuller, M. T. (2017). The conserved RNA helicase YTHDC2 regulates the transition from proliferation to differentiation in the germline. *eLife* 6, e26116. 10.7554/eLife.26116 29087293 PMC5703642

[DEV204227C3] Baker, S. M., Plug, A. W., Prolla, T. A., Bronner, C. E., Harris, A. C., Yao, X., Christie, D.-M., Monell, C., Arnheim, N., Bradley, A. et al. (1996). Involvement of mouse Mlh1 in DNA mismatch repair and meiotic crossing over. *Nat. Genet.* 13, 336-342. 10.1038/ng0796-336 8673133

[DEV204227C4] Baltus, A. E., Menke, D. B., Hu, Y.-C. C., Goodheart, M. L., Carpenter, A. E., De Rooij, D. G. and Page, D. C. (2006). In germ cells of mouse embryonic ovaries, the decision to enter meiosis precedes premeiotic DNA replication. *Nat. Genet.* 38, 1430-1434. 10.1038/ng1919 17115059

[DEV204227C5] Bannister, L. A., Reinholdt, L. G., Munroe, R. J. and Schimenti, J. C. (2004). Positional cloning and characterization of mouse mei8, a disrupted allelle of the meiotic cohesin Rec8. *Genesis* 40, 184-194. 10.1002/gene.20085 15515002

[DEV204227C6] Baudat, F., Manova, K., Yuen, J. P., Jasin, M. and Keeney, S. (2000). Chromosome synapsis defects and sexually dimorphic meiotic progression in mice lacking Spo11. *Mol. Cell* 6, 989-998. 10.1016/S1097-2765(00)00098-8 11106739

[DEV204227C7] Baudat, F., Buard, J., Grey, C., Fledel-Alon, A., Ober, C., Przeworski, M., Coop, G. and De Massy, B. (2010). PRDM9 is a major determinant of meiotic recombination hotspots in humans and mice. *Science* 327, 836-840. 10.1126/science.1183439 20044539 PMC4295902

[DEV204227C8] Bayne, R. A., Donnachie, D. J., Kinnell, H. L., Childs, A. J. and Anderson, R. A. (2016). BMP signalling in human fetal ovary somatic cells is modulated in a gene-specific fashion by GREM1 and GREM2. *Mol. Hum. Reprod.* 22, 622-633. 10.1093/molehr/gaw044 27385727 PMC5013871

[DEV204227C9] Bolcun-Filas, E., Hall, E., Speed, R., Taggart, M., Grey, C., De Massy, B., Benavente, R. and Cooke, H. J. (2009). Mutation of the mouse Syce1 gene disrupts synapsis and suggests a link between synaptonemal complex structural components and DNA repair. *PLoS Genet.* 5, e1000393. 10.1371/journal.pgen.1000393 19247432 PMC2640461

[DEV204227C10] Bolger, A. M., Lohse, M. and Usadel, B. (2014). Trimmomatic: a flexible trimmer for Illumina sequence data. *Bioinformatics* 30, 2114-2120. 10.1093/bioinformatics/btu170 24695404 PMC4103590

[DEV204227C11] Bowles, J., Knight, D., Smith, C., Wilhelm, D., Richman, J., Mamiya, S., Yashiro, K., Chawengsaksophak, K., Wilson, M. J., Rossant, J. et al. (2006). Retinoid signaling determines germ cell fate in mice. *Science* 312, 596-600. 10.1126/science.1125691 16574820

[DEV204227C12] Bowles, J., Feng, C.-W., Spiller, C., Davidson, T.-L., Jackson, A. and Koopman, P. (2010). FGF9 suppresses meiosis and promotes male germ cell fate in mice. *Dev. Cell* 19, 440-449. 10.1016/j.devcel.2010.08.010 20833365

[DEV204227C13] Bowles, J., Feng, C.-W., Miles, K., Ineson, J., Spiller, C. and Koopman, P. (2016). ALDH1A1 provides a source of meiosis-inducing retinoic acid in mouse fetal ovaries. *Nat. Commun.* 7, 10845-10845. 10.1038/ncomms10845 26892828 PMC4762892

[DEV204227C14] Bullejos, M. and Koopman, P. (2004). Germ cells enter meiosis in a rostro-caudal wave during development of the mouse ovary. *Mol. Reprod. Dev.* 68, 422-428. 10.1002/mrd.20105 15236325

[DEV204227C15] Burnet, G., Feng, C. W. A., Cheung, K. M. F., Bowles, J. and Spiller, C. M. (2023). Generation and characterization of a Ddx4-iCre transgenic line for deletion in the germline beginning at genital ridge colonization. *Genesis* 61, e23511. 10.1002/dvg.23511 36693128

[DEV204227C16] Chassot, A.-A., Le Rolle, M., Jolivet, G., Stevant, I., Guigonis, J.-M., Da Silva, F., Nef, S., Pailhoux, E., Schedl, A., Ghyselinck, N. B. et al. (2020). Retinoic acid synthesis by ALDH1A proteins is dispensable for meiosis initiation in the mouse fetal ovary. *Sci. Adv.* 6, eaaz1261. 10.1126/sciadv.aaz1261 32494737 PMC7244317

[DEV204227C17] Cheng, Y., Buffone, M. G., Kouadio, M., Goodheart, M., Page, D. C., Gerton, G. L., Davidson, I. and Wang, P. J. (2007). Abnormal sperm in mice lacking the Taf7l gene. *Mol. Cell. Biol.* 27, 2582-2589. 10.1128/MCB.01722-06 17242199 PMC1899882

[DEV204227C18] Chin, L., Pomerantz, J. and Depinho, R. A. (1998). The INK4a/ARF tumor suppressor: one gene--two products--two pathways. *Trends Biochem. Sci.* 23, 291-296. 10.1016/S0968-0004(98)01236-5 9757829

[DEV204227C19] Chuma, S. and Nakatsuji, N. (2001). Autonomous transition into meiosis of mouse fetal germ cells in vitro and its inhibition by gp130-mediated signaling. *Dev. Biol.* 229, 468-479. 10.1006/dbio.2000.9989 11203703

[DEV204227C20] Churchman, M. L., Roig, I., Jasin, M., Keeney, S. and Sherr, C. J. (2011). Expression of arf tumor suppressor in spermatogonia facilitates meiotic progression in male germ cells. *PLoS Genet.* 7, e1002157. 10.1371/journal.pgen.1002157 21811412 PMC3141002

[DEV204227C21] Cuny, G. D., Yu, P. B., Laha, J. K., Xing, X., Liu, J.-F., Lai, C. S., Deng, D. Y., Sachidanandan, C., Bloch, K. D. and Peterson, R. T. (2008). Structure-activity relationship study of bone morphogenetic protein (BMP) signaling inhibitors. *Bioorg. Med. Chem. Lett.* 18, 4388-4392. 10.1016/j.bmcl.2008.06.052 18621530 PMC2570262

[DEV204227C22] de Vries, S. S., Baart, E. B., Dekker, M., Siezen, A., De Rooij, D. G., De Boer, P. and Te Riele, H. (1999). Mouse MutS-like protein Msh5 is required for proper chromosome synapsis in male and female meiosis. *Genes Dev.* 13, 523-531. 10.1101/gad.13.5.523 10072381 PMC316502

[DEV204227C23] de Vries, F. A. T., de Boer, E., van den Bosch, M., Baarends, W. M., Ooms, M., Yuan, L., Liu, J.-G., van Zeeland, A. A., Heyting, C. and Pastink, A. (2005). Mouse Sycp1 functions in synaptonemal complex assembly, meiotic recombination, and XY body formation. *Genes Dev.* 19, 1376-1389. 10.1101/gad.329705 15937223 PMC1142560

[DEV204227C24] Diederichs, S., Bäumer, N., Schultz, N., Hamra, F. K., Schrader, M. G., Sandstede, M. L., Berdel, W. E., Serve, H. and Müller-Tidow, C. (2005). Expression patterns of mitotic and meiotic cell cycle regulators in testicular cancer and development. *Int. J. Cancer* 116, 207-217. 10.1002/ijc.21034 15800920

[DEV204227C25] Ding, X., Xu, R., Yu, J., Xu, T., Zhuang, Y. and Han, M. (2007). SUN1 is required for telomere attachment to nuclear envelope and gametogenesis in mice. *Dev. Cell* 12, 863-872. 10.1016/j.devcel.2007.03.018 17543860

[DEV204227C26] Dobin, A., Davis, C. A., Schlesinger, F., Drenkow, J., Zaleski, C., Jha, S., Batut, P., Chaisson, M. and Gingeras, T. R. (2013). STAR: ultrafast universal RNA-seq aligner. *Bioinformatics* 29, 15-21. 10.1093/bioinformatics/bts635 23104886 PMC3530905

[DEV204227C27] Dokshin, G. A., Baltus, A. E., Eppig, J. J. and Page, D. C. (2013). Oocyte differentiation is genetically dissociable from meiosis in mice. *Nat. Genet.* 45, 877-883. 10.1038/ng.2672 23770609 PMC3747777

[DEV204227C28] Dong, J., Albertini, D. F., Nishimori, K., Kumar, T. R., Lu, N. and Matzuk, M. M. (1996). Growth differentiation factor-9 is required during early ovarian folliculogenesis. *Nature* 383, 531-535. 10.1038/383531a0 8849725

[DEV204227C29] Dudley, B. M., Runyan, C., Takeuchi, Y., Schaible, K. and Molyneaux, K. (2007). BMP signaling regulates PGC numbers and motility in organ culture. *Mech. Dev.* 124, 68-77. 10.1016/j.mod.2006.09.005 17112707

[DEV204227C30] Feng, C.-W., Burnet, G., Spiller, C. M., Cheung, F. K. M., Chawengsaksophak, K., Koopman, P. and Bowles, J. (2021). Identification of regulatory elements required for Stra8 expression in fetal ovarian germ cells of the mouse. *Development* 148, dev194977. 10.1242/dev.194977 33574039

[DEV204227C31] Finsterbusch, F., Ravindranathan, R., Dereli, I., Stanzione, M., Tränkner, D. and Tóth, A. (2016). Alignment of homologous chromosomes and effective repair of programmed DNA double-strand breaks during mouse meiosis require the minichromosome maintenance domain containing 2 (MCMDC2) Protein. *PLoS Genet.* 12, e1006393. 10.1371/journal.pgen.1006393 27760146 PMC5070785

[DEV204227C32] Garrido-Martín, D., Palumbo, E., Guigó, R. and Breschi, A. (2018). ggsashimi: Sashimi plot revised for browser- and annotation-independent splicing visualization. *PLoS Comput. Biol.* 14, e1006360. 10.1371/journal.pcbi.1006360 30118475 PMC6114895

[DEV204227C33] Gill, M. E., Hu, Y.-C., Lin, Y. and Page, D. C. (2011). Licensing of gametogenesis, dependent on RNA binding protein DAZL, as a gateway to sexual differentiation of fetal germ cells. *Proc. Natl. Acad. Sci. USA* 108, 7443-7448. 10.1073/pnas.1104501108 21504946 PMC3088606

[DEV204227C34] Greder, L. V., Gupta, S., Li, S., Abedin, M. J., Sajini, A., Segal, Y., Slack, J. M. W. and Dutton, J. R. (2012). Analysis of endogenous Oct4 activation during induced pluripotent stem cell reprogramming using an inducible Oct4 lineage label. *Stem Cells* 30, 2596-2601. 10.1002/stem.1216 22948941 PMC3626284

[DEV204227C35] Grive, K. J., Seymour, K. A., Mehta, R. and Freiman, R. N. (2014). TAF4b promotes mouse primordial follicle assembly and oocyte survival. *Dev. Biol.* 392, 42-51. 10.1016/j.ydbio.2014.05.001 24836512 PMC4120270

[DEV204227C36] Gromley, A., Churchman, M. L., Zindy, F. and Sherr, C. J. (2009). Transient expression of the Arf tumor suppressor during male germ cell and eye development in Arf-Cre reporter mice. *Proc. Natl. Acad. Sci. USA* 106, 6285-6290. 10.1073/pnas.0902310106 19339492 PMC2664155

[DEV204227C37] Guiraldelli, M. F., Eyster, C., Wilkerson, J. L., Dresser, M. E. and Pezza, R. J. (2013). Mouse HFM1/Mer3 is required for crossover formation and complete synapsis of homologous chromosomes during meiosis. *PLoS Genet.* 9, e1003383. 10.1371/journal.pgen.1003383 23555294 PMC3605105

[DEV204227C38] Hajkova, P. (2010). Epigenetic reprogramming--taking a lesson from the embryo. *Curr. Opin. Cell Biol.* 22, 342-350. 10.1016/j.ceb.2010.04.011 20537882

[DEV204227C39] Hajkova, P., Erhardt, S., Lane, N., Haaf, T., El-Maarri, O., Reik, W., Walter, J. and Surani, M. A. (2002). Epigenetic reprogramming in mouse primordial germ cells. *Mech. Dev.* 117, 15-23. 10.1016/S0925-4773(02)00181-8 12204247

[DEV204227C40] Hamer, G., Wang, H., Bolcun-Filas, E., Cooke, H. J., Benavente, R. and Höög, C. (2008). Progression of meiotic recombination requires structural maturation of the central element of the synaptonemal complex. *J. Cell Sci.* 121, 2445-2451. 10.1242/jcs.033233 18611960

[DEV204227C41] Hayashi, K., Yoshida, K. and Matsui, Y. (2005). A histone H3 methyltransferase controls epigenetic events required for meiotic prophase. *Nature* 438, 374-378. 10.1038/nature04112 16292313

[DEV204227C42] Heaney, J. D., Anderson, E. L., Michelson, M. V., Zechel, J. L., Conrad, P. A., Page, D. C. and Nadeau, J. H. (2012). Germ cell pluripotency, premature differentiation and susceptibility to testicular teratomas in mice. *Development* 139, 1577-1586. 10.1242/dev.076851 22438569 PMC3317965

[DEV204227C43] Herrán, Y., Gutiérrez-Caballero, C., Sánchez-Martín, M., Hernández, T., Viera, A., Barbero, J. L., De Álava, E., de Rooij, D. G., Suja, J. A., Llano, E. et al. (2011). The cohesin subunit RAD21L functions in meiotic synapsis and exhibits sexual dimorphism in fertility. *EMBO J.* 30, 3091-3105. 10.1038/emboj.2011.222 21743440 PMC3160193

[DEV204227C44] Holloway, J. K., Sun, X., Yokoo, R., Villeneuve, A. M. and Cohen, P. E. (2014). Mammalian CNTD1 is critical for meiotic crossover maturation and deselection of excess precrossover sites. *J. Cell Biol.* 205, 633-641. 10.1083/jcb.201401122 24891606 PMC4050721

[DEV204227C45] Hopkins, J., Hwang, G., Jacob, J., Sapp, N., Bedigian, R., Oka, K., Overbeek, P., Murray, S. and Jordan, P. W. (2014). Meiosis-specific cohesin component, Stag3 is essential for maintaining centromere chromatid cohesion, and required for DNA repair and synapsis between homologous chromosomes. *PLoS Genet.* 10, e1004413. 10.1371/journal.pgen.1004413 24992337 PMC4081007

[DEV204227C46] Horn, H. F., Kim, D. I., Wright, G. D., Wong, E. S. M., Stewart, C. L., Burke, B. and Roux, K. J. (2013). A mammalian KASH domain protein coupling meiotic chromosomes to the cytoskeleton. *J. Cell Biol.* 202, 1023-1039. 10.1083/jcb.201304004 24062341 PMC3787381

[DEV204227C47] Huang, D. W., Sherman, B. T. and Lempicki, R. A. (2009). Systematic and integrative analysis of large gene lists using DAVID bioinformatics resources. *Nat. Protoc.* 4, 44-57. 10.1038/nprot.2008.211 19131956

[DEV204227C48] Hwang, G. H., Hopkins, J. L. and Jordan, P. W. (2018). Chromatin spread preparations for the analysis of mouse oocyte progression from prophase to metaphase II. *J. Vis. Exp.* 132, 56736. 10.3791/56736 PMC5931380 29553540

[DEV204227C49] Ishiguro, K., Kim, J., Fujiyama-Nakamura, S., Kato, S. and Watanabe, Y. (2011). A new meiosis-specific cohesin complex implicated in the cohesin code for homologous pairing. *EMBO Rep.* 12, 267-275. 10.1038/embor.2011.2 21274006 PMC3059921

[DEV204227C50] Ishiguro, K.-I., Matsuura, K., Tani, N., Takeda, N., Usuki, S., Yamane, M., Sugimoto, M., Fujimura, S., Hosokawa, M., Chuma, S. et al. (2020). MEIOSIN directs the switch from mitosis to kmeiosis in mammalian germ cells. *Dev. Cell* 52, 429-445.e410. 10.1016/j.devcel.2020.01.010 32032549

[DEV204227C51] Jameson, S. A., Natarajan, A., Cool, J., Defalco, T., Maatouk, D. M., Mork, L., Munger, S. C. and Capel, B. (2012). Temporal transcriptional profiling of somatic and germ cells reveals biased lineage priming of sexual fate in the fetal mouse gonad. *PLoS Genet.* 8, e1002575. 10.1371/journal.pgen.1002575 22438826 PMC3305395

[DEV204227C52] Kashimada, K., Pelosi, E., Chen, H., Schlessinger, D., Wilhelm, D. and Koopman, P. (2011). FOXL2 and BMP2 act cooperatively to regulate follistatin gene expression during ovarian development. *Endocrinology* 152, 272-280. 10.1210/en.2010-0636 21084449 PMC3219046

[DEV204227C53] Keeney, S., Giroux, C. N. and Kleckner, N. (1997). Meiosis-specific DNA double-strand breaks are catalyzed by Spo11, a member of a widely conserved protein family. *Cell* 88, 375-384. 10.1016/S0092-8674(00)81876-0 9039264

[DEV204227C54] Kimble, J. (2011). Molecular regulation of the mitosis/meiosis decision in multicellular organisms. *Cold Spring Harb. Perspect. Biol.* 3, a002683. 10.1101/cshperspect.a002683 21646377 PMC3140684

[DEV204227C55] Kneitz, B., Cohen, P. E., Avdievich, E., Zhu, L., Kane, M. F., Hou, H., Jr, Kolodner, R. D., Kucherlapati, R., Pollard, J. W. and Edelmann, W. (2000). MutS homolog 4 localization to meiotic chromosomes is required for chromosome pairing during meiosis in male and female mice. *Genes Dev.* 14, 1085-1097. 10.1101/gad.14.9.1085 10809667 PMC316572

[DEV204227C56] Kogo, H., Tsutsumi, M., Inagaki, H., Ohye, T., Kiyonari, H. and Kurahashi, H. (2012). HORMAD2 is essential for synapsis surveillance during meiotic prophase via the recruitment of ATR activity. *Genes Cells* 17, 897-912. 10.1111/gtc.12005 23039116

[DEV204227C57] Kojima, M. L., De Rooij, D. G. and Page, D. C. (2019). Amplification of a broad transcriptional program by a common factor triggers the meiotic cell cycle in mice. *eLife* 8, e43738. 10.7554/eLife.43738.057 30810530 PMC6392498

[DEV204227C58] Koubova, J., Menke, D. B., Zhou, Q., Capel, B., Griswold, M. D., Page, D. C., Cape, B., Griswold, M. D. and Page, D. C. (2006). Retinoic acid regulates sex-specific timing of meiotic initiation in mice. *Proc. Natl. Acad. Sci. USA* 103, 2474-2479. 10.1073/pnas.0510813103 16461896 PMC1413806

[DEV204227C59] Koubova, J., Hu, Y.-C., Bhattacharyya, T., Soh, Y. Q. S., Gill, M. E., Goodheart, M. L., Hogarth, C. A., Griswold, M. D. and Page, D. C. (2014). Retinoic acid activates two pathways required for meiosis in mice. *PLoS Genet.* 10, e1004541. 10.1371/journal.pgen.1004541 25102060 PMC4125102

[DEV204227C60] Krentz, A. D., Murphy, M. W., Sarver, A. L., Griswold, M. D., Bardwell, V. J. and Zarkower, D. (2011). DMRT1 promotes oogenesis by transcriptional activation of Stra8 in the mammalian fetal ovary. *Dev. Biol.* 356, 63-70. 10.1016/j.ydbio.2011.05.658 21621532 PMC3131262

[DEV204227C61] Kumar, R., Bourbon, H.-M. and de Massy, B. (2010). Functional conservation of Mei4 for meiotic DNA double-strand break formation from yeasts to mice. *Genes Dev.* 24, 1266-1280. 10.1101/gad.571710 20551173 PMC2885662

[DEV204227C62] Kumar, S., Chatzi, C., Brade, T., Cunningham, T. J., Zhao, X. and Duester, G. (2011). Sex-specific timing of meiotic initiation is regulated by Cyp26b1 independent of retinoic acid signalling. *Nat. Commun.* 2, 151. 10.1038/ncomms1136 21224842 PMC3034736

[DEV204227C63] La Salle, S., Palmer, K., O'Brien, M., Schimenti, J. C., Eppig, J. and Handel, M. A. (2012). Spata22, a novel vertebrate-specific gene, is required for meiotic progress in mouse germ cells. *Biol. Reprod.* 86, 45. 10.1095/biolreprod.111.095752 22011390 PMC3290669

[DEV204227C64] Lawson, K. A., Dunn, N. R., Roelen, B. A. J., Zeinstra, L. M., Davis, A. M., Wright, C. V. E., Korving, J. P. W. F. M. and Hogan, B. L. M. (1999). Bmp4 is required for the generation of primordial germ cells in the mouse embryo. *Genes Dev.* 13, 424-436. 10.1101/gad.13.4.424 10049358 PMC316469

[DEV204227C65] Lee, J. and Hirano, T. (2011). RAD21L, a novel cohesin subunit implicated in linking homologous chromosomes in mammalian meiosis. *J. Cell Biol.* 192, 263-276. 10.1083/jcb.201008005 21242291 PMC3172173

[DEV204227C66] Li, Y., Wu, Y., Khan, I., Zhou, J., Lu, Y., Ye, J., Liu, J., Xie, X., Hu, C., Jiang, H. et al. (2023). M1AP interacts with the mammalian ZZS complex and promotes male meiotic recombination. *EMBO Rep.* 24, e55778. 10.15252/embr.202255778 36440627 PMC9900333

[DEV204227C67] Libby, B. J., Reinholdt, L. G. and Schimenti, J. C. (2003). Positional cloning and characterization of Mei1, a vertebrate-specific gene required for normal meiotic chromosome synapsis in mice. *Proc. Natl. Acad. Sci. USA* 100, 15706-15711. 10.1073/pnas.2432067100 14668445 PMC307632

[DEV204227C68] Lim, C. H., Brower, J. V., Resnick, J. L., Oh, S. P. and Terada, N. (2015). Adenine nucleotide translocase 4 is expressed within embryonic ovaries and dispensable during oogenesis. *Reprod. Sci.* 22, 250-257. 10.1177/1933719114542026 25031318 PMC4287603

[DEV204227C69] MacLean, G., Li, H., Metzger, D., Chambon, P. and Petkovich, M. (2007). Apoptotic extinction of germ cells in testes of Cyp26b1 knockout mice. *Endocrinology* 148, 4560-4567. 10.1210/en.2007-0492 17584971

[DEV204227C70] Martinerie, L., Manterola, M., Chung, S. S. W., Panigrahi, S. K., Weisbach, M., Vasileva, A., Geng, Y., Sicinski, P. and Wolgemuth, D. J. (2014). Mammalian E-type cyclins control chromosome pairing, telomere stability and CDK2 localization in male meiosis. *PLoS Genet.* 10, e1004165. 10.1371/journal.pgen.1004165 24586195 PMC3937215

[DEV204227C71] McClelland, K. S. and Bowles, J. (2016). Culturing murine embryonic organs: Pros, cons, tips and tricks. *Differentiation* 91, 50-56. 10.1016/j.diff.2016.01.008 26988290

[DEV204227C72] McLaren, A. (1995). Germ cells and germ cell sex. *Philos. Trans. R. Soc. Lond. Ser. B Biol. Sci.* 350, 229-233. 10.1098/rstb.1995.0156 8570686

[DEV204227C73] McLaren, A. (2003). Primordial germ cells in the mouse. *Dev. Biol.* 262, 1-15. 10.1016/S0012-1606(03)00214-8 14512014

[DEV204227C74] Medani, H., Elshiekh, M. and Naresh, K. N. (2021). Improving precise counting of mitotic cells in mantle cell lymphoma using phosphohistone H3 (PHH3) antibody. *J. Clin. Pathol.* 74, 646-649. 10.1136/jclinpath-2020-206956 32873701

[DEV204227C75] Menke, D. B., Koubova, J. and Page, D. C. (2003). Sexual differentiation of germ cells in XX mouse gonads occurs in an anterior-to-posterior wave. *Dev. Biol.* 262, 303-312. 10.1016/S0012-1606(03)00391-9 14550793

[DEV204227C76] Mishina, Y., Hanks, M. C., Miura, S., Tallquist, M. D. and Behringer, R. R. (2002). Generation of Bmpr/Alk3 conditional knockout mice. *Genesis (New York, N.Y. : 2000)* 32, 69-72. 10.1002/gene.10038 11857780

[DEV204227C77] Miyauchi, H., Ohta, H., Nagaoka, S., Nakaki, F., Sasaki, K., Hayashi, K., Yabuta, Y., Nakamura, T., Yamamoto, T. and Saitou, M. (2017). Bone morphogenetic protein and retinoic acid synergistically specify female germ-cell fate in mice. *EMBO J.* 36, 3100-3119. 10.15252/embj.201796875 28928204 PMC5666620

[DEV204227C78] Mueller, T. D. and Nickel, J. (2012). Promiscuity and specificity in BMP receptor activation. *FEBS Lett.* 586, 1846-1859. 10.1016/j.febslet.2012.02.043 22710174

[DEV204227C79] Nagaoka, S. I., Nakaki, F., Miyauchi, H., Nosaka, Y., Ohta, H., Yabuta, Y., Kurimoto, K., Hayashi, K., Nakamura, T., Yamamoto, T. et al. (2020). ZGLP1 is a determinant for the oogenic fate in mice. *Science* 367, eaaw4115. 10.1126/science.aaw4115 32054698

[DEV204227C80] Ohinata, Y., Ohta, H., Shigeta, M., Yamanaka, K., Wakayama, T. and Saitou, M. (2009). A signaling principle for the specification of the germ cell lineage in mice. *Cell* 137, 571-584. 10.1016/j.cell.2009.03.014 19410550

[DEV204227C81] Ohta, H., Kurimoto, K., Okamoto, I., Nakamura, T., Yabuta, Y., Miyauchi, H., Yamamoto, T., Okuno, Y., Hagiwara, M., Shirane, K. et al. (2017). In vitro expansion of mouse primordial germ cell-like cells recapitulates an epigenetic blank slate. *EMBO J.* 36, 1888-1907. 10.15252/embj.201695862 28559416 PMC5494472

[DEV204227C82] Pangas, S. A., Li, X., Robertson, E. J. and Matzuk, M. M. (2006). Premature luteinization and cumulus cell defects in ovarian-specific Smad4 knockout mice. *Mol. Endocrinol.* 20, 1406-1422. 10.1210/me.2005-0462 16513794

[DEV204227C83] Papanikos, F., Clement, J. A. J., Testa, E., Ravindranathan, R., Grey, C., Dereli, I., Bondarieva, A., Valerio-Cabrera, S., Stanzione, M., Schleiffer, A. et al. (2019). Mouse ANKRD31 regulates spatiotemporal patterning of meiotic recombination initiation and ensures recombination between X and Y sex chromosomes. *Mol. Cell* 74, 1069-1085.e1011. 10.1016/j.molcel.2019.03.022 31000436

[DEV204227C84] Pesce, M., Wang, X., Wolgemuth, D. J. and Schöler, H. (1998). Differential expression of the Oct-4 transcription factor during mouse germ cell differentiation. *Mech. Dev.* 71, 89-98. 10.1016/S0925-4773(98)00002-1 9507072

[DEV204227C85] Pittman, D. L., Cobb, J., Schimenti, K. J., Wilson, L. A., Cooper, D. M., Brignull, E., Handel, M. A. and Schimenti, J. C. (1998). Meiotic prophase arrest with failure of chromosome synapsis in mice deficient for Dmc1, a germline-specific RecA homolog. *Mol. Cell* 1, 697-705. 10.1016/S1097-2765(00)80069-6 9660953

[DEV204227C86] Prieto, I., Tease, C., Pezzi, N., Buesa, J. M., Ortega, S., Kremer, L., Martinez, A., Martínez, A. C., Hultén, M. A. and Barbero, J. L. (2004). Cohesin component dynamics during meiotic prophase I in mammalian oocytes. *Chromosome Res.* 12, 197-213. 10.1023/B:CHRO.0000021945.83198.0e 15125634

[DEV204227C87] Revenkova, E., Eijpe, M., Heyting, C., Hodges, C. A., Hunt, P. A., Liebe, B., Scherthan, H. and Jessberger, R. (2004). Cohesin SMC1 beta is required for meiotic chromosome dynamics, sister chromatid cohesion and DNA recombination. *Nat. Cell Biol.* 6, 555-562. 10.1038/ncb1135 15146193

[DEV204227C88] Reynolds, A., Qiao, H., Yang, Y., Chen, J. K., Jackson, N., Biswas, K., Holloway, J. K., Baudat, F., De Massy, B., Wang, J. et al. (2013). RNF212 is a dosage-sensitive regulator of crossing-over during mammalian meiosis. *Nat. Genet.* 45, 269-278. 10.1038/ng.2541 23396135 PMC4245152

[DEV204227C89] Robert, T., Nore, A., Brun, C., Maffre, C., Crimi, B., Bourbon, H.-M. and de Massy, B. (2016). The TopoVIB-Like protein family is required for meiotic DNA double-strand break formation. *Science* 351, 943-949. 10.1126/science.aad5309 26917764

[DEV204227C90] Robinson, M. D., McCarthy, D. J. and Smyth, G. K. (2010). edgeR: a Bioconductor package for differential expression analysis of digital gene expression data. *Bioinformatics* 26, 139-140. 10.1093/bioinformatics/btp616 19910308 PMC2796818

[DEV204227C91] Ross, A., Munger, S. and Capel, B. (2007). Bmp7 regulates germ cell proliferation in mouse fetal gonads. *Sex. Dev.* 1, 127-137. 10.1159/000100034 18391523

[DEV204227C92] Saitou, M., Barton, S. C. and Surani, M. A. (2002). A molecular programme for the specification of germ cell fate in mice. *Nature* 418, 293-300. 10.1038/nature00927 12124616

[DEV204227C93] Savery, D., Maniou, E., Culshaw, L. H., Greene, N. D. E., Copp, A. J. and Galea, G. L. (2020). Refinement of inducible gene deletion in embryos of pregnant mice. *Birth Defects Res.* 112, 196-204. 10.1002/bdr2.1628 31793758 PMC7003956

[DEV204227C94] Schramm, S., Fraune, J., Naumann, R., Hernandez-Hernandez, A., Höög, C., Cooke, H. J., Alsheimer, M. and Benavente, R. (2011). A novel mouse synaptonemal complex protein is essential for loading of central element proteins, recombination, and fertility. *PLoS Genet.* 7, e1002088. 10.1371/journal.pgen.1002088 21637789 PMC3102746

[DEV204227C95] Seligman, J. and Page, D. C. (1998). The Dazh gene is expressed in male and female embryonic gonads before germ cell sex differentiation. *Biochem. Biophys. Res. Commun.* 245, 878-882. 10.1006/bbrc.1998.8530 9588208

[DEV204227C96] Sherman, B. T., Hao, M., Qiu, J., Jiao, X., Baseler, M. W., Lane, H. C., Imamichi, T. and Chang, W. (2022). DAVID: a web server for functional enrichment analysis and functional annotation of gene lists (2021 update). *Nucleic Acids Res.* 50, W216-W221. 10.1093/nar/gkac194 35325185 PMC9252805

[DEV204227C97] Shibuya, H., Ishiguro, K. and Watanabe, Y. (2014). The TRF1-binding protein TERB1 promotes chromosome movement and telomere rigidity in meiosis. *Nat. Cell Biol.* 16, 145-156. 10.1038/ncb2896 24413433

[DEV204227C98] Shibuya, H., Hernández-Hernández, A., Morimoto, A., Negishi, L., Höög, C. and Watanabe, Y. (2015). MAJIN links telomeric DNA to the nuclear membrane by exchanging telomere cap. *Cell* 163, 1252-1266. 10.1016/j.cell.2015.10.030 26548954

[DEV204227C99] Shimada, R., Kato, Y., Takeda, N., Fujimura, S., Yasunaga, K.-I., Usuki, S., Niwa, H., Araki, K. and Ishiguro, K.-I. (2023). STRA8-RB interaction is required for timely entry of meiosis in mouse female germ cells. *Nat. Commun.* 14, 6443. 10.1038/s41467-023-42259-6 37880249 PMC10600341

[DEV204227C100] Shin, Y.-H., Choi, Y., Erdin, S. U., Yatsenko, S. A., Kloc, M., Yang, F., Wang, P. J., Meistrich, M. L. and Rajkovic, A. (2010). Hormad1 mutation disrupts synaptonemal complex formation, recombination, and chromosome segregation in mammalian meiosis. *PLoS Genet.* 6, e1001190. 10.1371/journal.pgen.1001190 21079677 PMC2973818

[DEV204227C101] Shin, Y.-H., Ren, Y., Suzuki, H., Golnoski, K. J., Ahn, H. W., Mico, V. and Rajkovic, A. (2017). Transcription factors SOHLH1 and SOHLH2 coordinate oocyte differentiation without affecting meiosis I. *J. Clin. Invest.* 127, 2106-2117. 10.1172/JCI90281 28504655 PMC5451230

[DEV204227C102] Soh, Y. Q. S., Junker, J. P., Gill, M. E., Mueller, J. L., Van Oudenaarden, A. and Page, D. C. (2015). A gene regulatory program for meiotic prophase in the fetal ovary. *PLoS Genet.* 11, e1005531. 10.1371/journal.pgen.1005531 26378784 PMC4574967

[DEV204227C103] Soh, Y. Q. S., Mikedis, M. M., Kojima, M., Godfrey, A. K., De Rooij, D. G. and Page, D. C. (2017). Meioc maintains an extended meiotic prophase I in mice. *PLoS Genet.* 13, e1006704. 10.1371/journal.pgen.1006704 28380054 PMC5397071

[DEV204227C104] Souquet, B., Abby, E., Hervé, R., Finsterbusch, F., Tourpin, S., Le Bouffant, R., Duquenne, C., Messiaen, S., Martini, E., Bernardino-Sgherri, J. et al. (2013). MEIOB targets single-strand DNA and is necessary for meiotic recombination. *PLoS Genet.* 9, e1003784. 10.1371/journal.pgen.1003784 24068956 PMC3778009

[DEV204227C105] Soyal, S. M., Amleh, A. and Dean, J. (2000). FIGalpha, a germ cell-specific transcription factor required for ovarian follicle formation. *Development* 127, 4645-4654. 10.1242/dev.127.21.4645 11023867

[DEV204227C106] Spiller, C. M., Feng, C.-W., Jackson, A., Gillis, A. J. M., Rolland, A. D., Looijenga, L. H. J., Koopman, P. and Bowles, J. (2012). Endogenous Nodal signaling regulates germ cell potency during mammalian testis development. *Development* 139, 4123-4132. 10.1242/dev.083006 23034635

[DEV204227C107] Stanzione, M., Baumann, M., Papanikos, F., Dereli, I., Lange, J., Ramlal, A., Tränkner, D., Shibuya, H., De Massy, B., Watanabe, Y. et al. (2016). Meiotic DNA break formation requires the unsynapsed chromosome axis-binding protein IHO1 (CCDC36) in mice. *Nat. Cell Biol.* 18, 1208-1220. 10.1038/ncb3417 27723721 PMC5089853

[DEV204227C108] Takemoto, K., Tani, N., Takada-Horisawa, Y., Fujimura, S., Tanno, N., Yamane, M., Okamura, K., Sugimoto, M., Araki, K. and Ishiguro, K.-I. (2020). Meiosis-specific C19orf57/4930432K21Rik/BRME1 modulates localization of RAD51 and DMC1 to DSBs in mouse meiotic recombination. *Cell Rep.* 31, 107686. 10.1016/j.celrep.2020.107686 32460033

[DEV204227C109] Tedesco, M., La Sala, G., Barbagallo, F., De Felici, M. and Farini, D. (2009). STRA8 shuttles between nucleus and cytoplasm and displays transcriptional activity. *J. Biol. Chem.* 284, 35781-35793. 10.1074/jbc.M109.056481 19805549 PMC2791008

[DEV204227C110] Toyooka, Y., Tsunekawa, N., Takahashi, Y., Matsui, Y., Satoh, M. and Noce, T. (2000). Expression and intracellular localization of mouse Vasa-homologue protein during germ cell development. *Mech. Dev.* 93, 139-149. 10.1016/S0925-4773(00)00283-5 10781947

[DEV204227C111] Vernet, N., Condrea, D., Mayere, C., Féret, B., Klopfenstein, M., Magnant, W., Alunni, V., Teletin, M., Souali-Crespo, S., Nef, S. et al. (2020). Meiosis occurs normally in the fetal ovary of mice lacking all retinoic acid receptors. *Sci. Adv.* 6, eaaz1139. 10.1126/sciadv.aaz1139 32917583 PMC7244263

[DEV204227C112] Western, P. S., Van Den Bergen, J. A., Miles, D. C. and Sinclair, A. H. (2010). Male fetal germ cell differentiation involves complex repression of the regulatory network controlling pluripotency. *FASEB J.* 24, 3026-3035. 10.1096/fj.09-151555 20395456

[DEV204227C113] Wojtasz, L., Cloutier, J. M., Baumann, M., Daniel, K., Varga, J., Fu, J., Anastassiadis, K., Stewart, A. F., Reményi, A., Turner, J. M. A. et al. (2012). Meiotic DNA double-strand breaks and chromosome asynapsis in mice are monitored by distinct HORMAD2-independent and -dependent mechanisms. *Genes Dev.* 26, 958-973. 10.1101/gad.187559.112 22549958 PMC3347793

[DEV204227C114] Xu, H., Beasley, M. D., Warren, W. D., van der Horst, G. T. and McKay, M. J. (2005). Absence of mouse REC8 cohesin promotes synapsis of sister chromatids in meiosis. *Dev. Cell* 8, 949-961. 10.1016/j.devcel.2005.03.018 15935783

[DEV204227C115] Yan, C., Wang, P., Demayo, J., Demayo, F. J., Elvin, J. A., Carino, C., Prasad, S. V., Skinner, S. S., Dunbar, B. S., Dube, J. L. et al. (2001). Synergistic roles of bone morphogenetic protein 15 and growth differentiation factor 9 in ovarian function. *Mol. Endocrinol.* 15, 854-866. 10.1210/mend.15.6.0662 11376106

[DEV204227C116] Yang, F., De La Fuente, R., Leu, N. A., Baumann, C., McLaughlin, K. J. and Wang, P. J. (2006). Mouse SYCP2 is required for synaptonemal complex assembly and chromosomal synapsis during male meiosis. *J. Cell Biol.* 173, 497-507. 10.1083/jcb.200603063 16717126 PMC2063860

[DEV204227C117] Yang, F., Gell, K., Van Der Heijden, G. W., Eckardt, S., Leu, N. A., Page, D. C., Benavente, R., Her, C., Höög, C., McLaughlin, K. J. et al. (2008). Meiotic failure in male mice lacking an X-linked factor. *Genes Dev.* 22, 682-691. 10.1101/gad.1613608 18316482 PMC2259036

[DEV204227C118] Yao, H. H. C., Matzuk, M. M., Jorgez, C. J., Menke, D. B., Page, D. C., Swain, A. and Capel, B. (2004). Follistatin operates downstream of Wnt4 in mammalian ovary organogenesis. *Dev. Dyn.* 230, 210-215. 10.1002/dvdy.20042 15162500 PMC4046253

[DEV204227C119] Ying, Y., Liu, X.-M., Marble, A., Lawson, K. A. and Zhao, G.-Q. (2000). Requirement of Bmp8b for the generation of primordial germ cells in the mouse. *Mol. Endocrinol.* 14, 1053-1063. 10.1210/mend.14.7.0479 10894154

[DEV204227C120] Ying, Y., Qi, X. and Zhao, G.-Q. (2001). Induction of primordial germ cells from murine epiblasts by synergistic action of BMP4 and BMP8B signaling pathways. *Proc. Natl. Acad. Sci. USA* 98, 7858-7862. 10.1073/pnas.151242798 11427739 PMC35432

[DEV204227C121] Yoshida, K., Kondoh, G., Matsuda, Y., Habu, T., Nishimune, Y. and Morita, T. (1998). The mouse RecA-like gene Dmc1 is required for homologous chromosome synapsis during meiosis. *Mol. Cell* 1, 707-718. 10.1016/S1097-2765(00)80070-2 9660954

[DEV204227C122] Yuan, L., Liu, J.-G., Hoja, M.-R., Wilbertz, J., Nordqvist, K. and Höög, C. (2002). Female germ cell aneuploidy and embryo death in mice lacking the meiosis-specific protein SCP3. *Science* 296, 1115-1118. 10.1126/science.1070594 12004129

[DEV204227C123] Zhang, H. and Bradley, A. (1996). Mice deficient for BMP2 are nonviable and have defects in amnion/chorion and cardiac development. *Development* 122, 2977-2986. 10.1242/dev.122.10.2977 8898212

[DEV204227C124] Zhao, Z. H., Ma, J. Y., Meng, T. G., Wang, Z. B., Yue, W., Zhou, Q., Li, S., Feng, X., Hou, Y., Schatten, H. et al. (2020). Single-cell RNA sequencing reveals the landscape of early female germ cell development. *FASEB J.* 34, 12634-12645. 10.1096/fj.202001034RR 32716582

