# Supplementary material for: BMP and STRA8 act collaboratively to ensure correct mitotic-to-meiotic transition in the fetal mouse ovary
Source: Development. 2025 Feb 7;152(3):DEV204227. doi: 10.1242/dev.204227 (PMC11829761; doi:10.1242/dev.204227)
Supplement: Supplementary information [file develop-152-204227-s1.pdf]

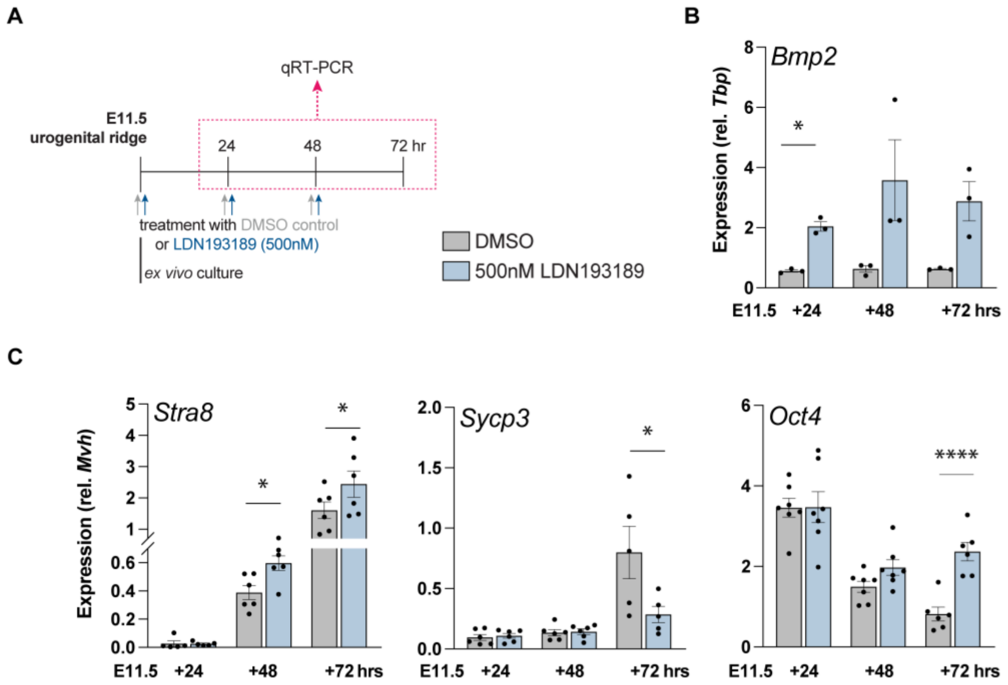

Fig. S1. Female urogenital ridges cultured *ex vivo* with BMP signalling antagonist LDN193189 showed delayed expression of meiotic genes and maintenance of pluripotency. (A) Urogenital ridges (UGRs) were dissected from E11.5 C57BL/6 female embryos and cultured *ex vivo* for 24, 48, or 72 hours in hanging drops with or without 500nM LDN193189. **(B)** LDN193189-treated UGRs significantly upregulated *Bmp2* expression after 24 hr culture. **(C)** Expression of *Stra8* in LDN193189-treated UGRs was significantly higher when cultured for 48 or 72 hr, whilst *Sycp3* expression was significantly lower in 72 hr-treated samples compared to the control. Treatment with LDN193189 for 72 hr also resulted in maintained expression of pluripotency marker *Oct4*. * $p < 0.05$, **** $p < 0.0001$ ($n \geq 4$, paired t-test, mean ± SEM).

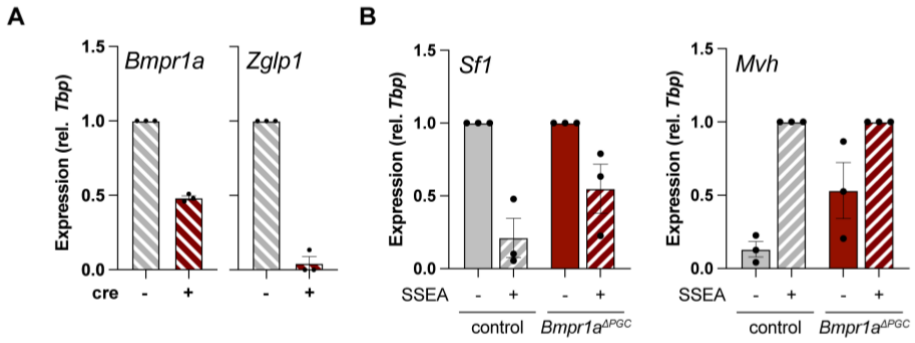

Fig. S2. Reduced expression of *Bmpr1a* in germ cell-enriched populations of *Bmpr1a*$^{\Delta PGC}$ ovaries. (A) To assess the specific and efficacy of the inducible knockout, germ cells were enriched from E13.5 *Bmpr1a*$^{\Delta PGC}$ and control ovaries by MACS using positive selection with anti-SSEA1 microbeads. *Bmpr1a*$^{\Delta PGC}$ cell population enriched for germ cells (SSEA1+) showed substantially reduced expression of *Bmpr1a* and low or no expression of the germ cell-specific BMP target *Zglp1* (Nagaoka et al., 2012), compared to the *Bmpr1a*$^{fl/fl}$;*Oct4-Cre*$^{WT/WT}$ control germ cell-enriched population. Expression levels of the control set to 1. **(B)** Purity of the enrichment was assessed using TaqMan qRT-PCR for the expression of somatic marker gene *Sf1* and germ cell-specific marker *Mvh*. Expression levels were normalised to *Tbp*. Expression level of *Sf1* in the SSEA- fraction was set to 1 and expression level of *Mvh* in the SSEA+ fraction was set to 1. Results were collected from 3 experimental replicates, each replicate was a pool of minimum 4 gonad pairs collected from one or two litters. The considerable somatic cell contamination in the SSEA+ fraction likely account for the remaining *Bmpr1a* expression in the mutant sample, as *Bmpr1a* is also highly expressed in gonadal somatic cells during this stage (Jameson et al, 2012).

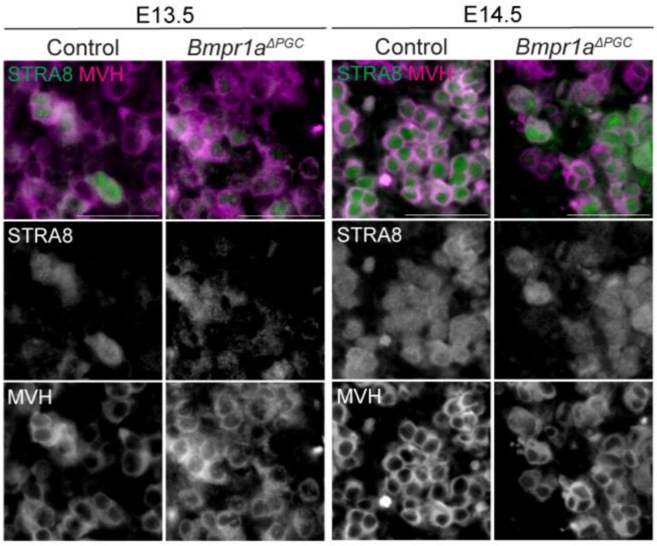

Fig. S3. Subcellular localisation of STRA8 was comparable between *Bmpr1a*$^{\Delta PGC}$ and control germ cells at E13.5 and E14.5. Immunofluorescence signal for STRA8 (green) was found in both the nucleus and cytoplasm of some MVH^{+} (magenta) germ cells, with no apparent skew towards the cytoplasm in *Bmpr1a*$^{\Delta PGC}$ germ cells. Scale bars represent 50 μm.

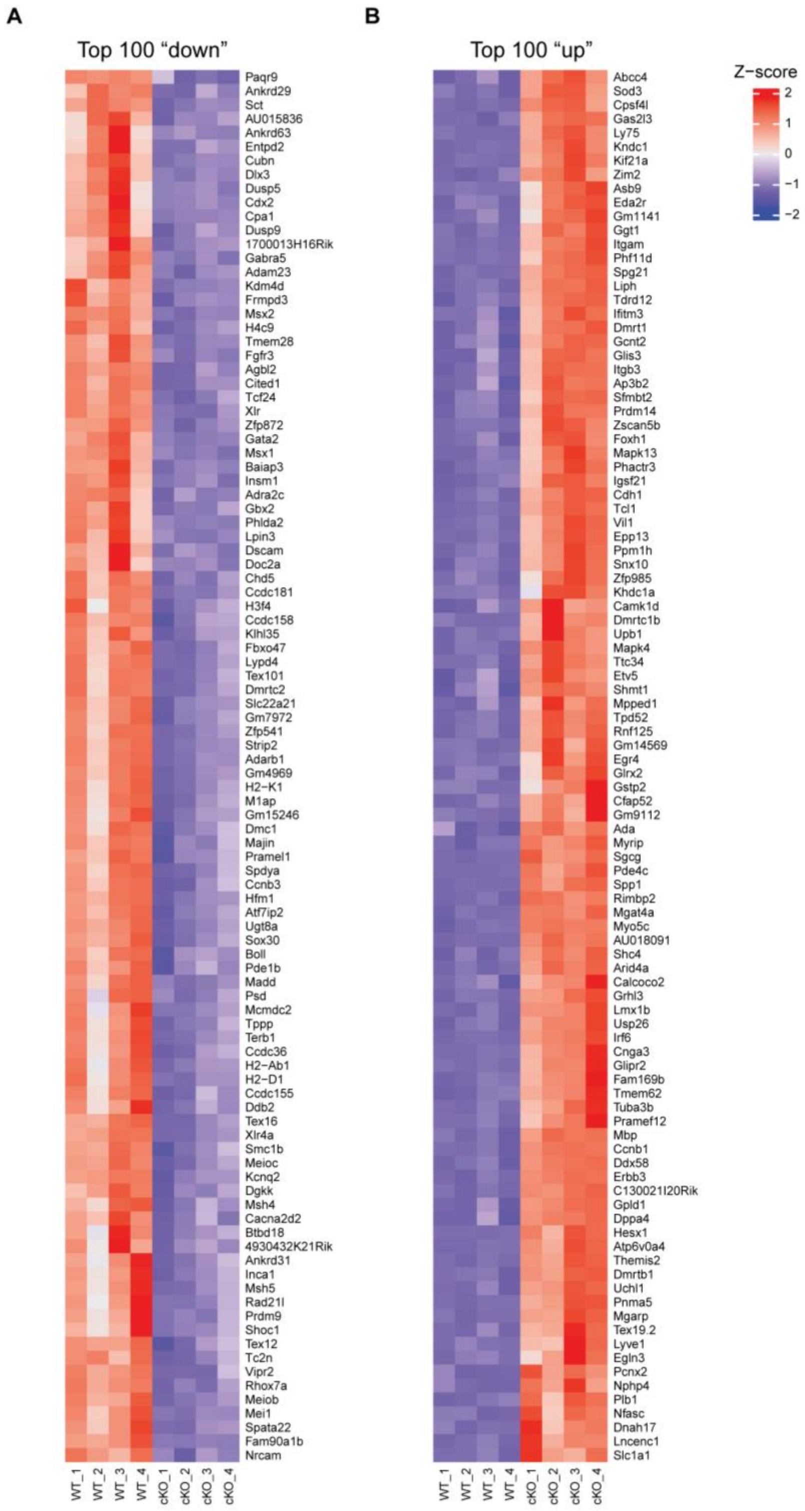

Fig. S4. Germ cell-specific loss of *Bmpr1a* resulted in aberrant gene expression in E14.5 fetal mouse ovaries. Top 100 differentially (A) downregulated or (B) upregulated genes in E14.5 *Bmpr1a*-cKO ovaries compared to the control. Differential gene expression was defined as those expressed with a |log2FC| > 0.5 and FDR < 0.05. Full list of differentially expressed genes is listed in **Table S1.**

Development • Supplementary information

Table S1. Differentially expressed genes between E14.5 *Bmpr1a*-cKO and *Bmpr1a*-flox control ovaries.

Available for download at
https://journals.biologists.com/dev/article-lookup/doi/10.1242/dev.204227#supplementary-data

Table S2. Enriched biological processes GO terms associated with the top 5 annotated clusters for differentially downregulated genes in *Bmpr1a*-cKO.

Available for download at
https://journals.biologists.com/dev/article-lookup/doi/10.1242/dev.204227#supplementary-data

Table S3. Enriched biological processes GO terms associated with the top 5 annotated clusters for differentially upregulated genes in *Bmpr1a*-cKO.

Available for download at
https://journals.biologists.com/dev/article-lookup/doi/10.1242/dev.204227#supplementary-data

Table S4. Differentially expressed genes in E14.5 *Bmpr1a*-cKO and/or *Stra8*-null ovaries.

Available for download at
https://journals.biologists.com/dev/article-lookup/doi/10.1242/dev.204227#supplementary-data

Table S5. Differential gene expression result for E14.5 *Stra8-null* vs WT control ovaries.

Available for download at
https://journals.biologists.com/dev/article-lookup/doi/10.1242/dev.204227#supplementary-data

Table S6. Genotyping PCR primer sequences.

Name	Sequence
Bmpr1a flox genotyping (MMRRC #030469):	
Bmpr1a-fl_fx2	GCAGCTGCTGCTGCAGCCTCC
Bmpr1a-fl_fx4	TGGCTACAATTTGTCTCATGC
Oct4-Cre genotyping (Greder et al., 2012):	
Pou-Cre_F	CCAAGGCAAGGGAGGTAGACAAG
Pou-Cre_WTR	GCTTTCTCCAACCGCAGGCTCTC
Pou-Cre_mutR	GCCCTCACATTGCCAAAAGACGG
Sex genotyping (Chuma and Nakatsuji, 2001):	
Ube-1x-1	TGGTCTGGACCCAAACGCTGTCCACA
Ube-1x-2	GGCAGCAGCCATCACATAATCCAGATG

Table S7. TaqMan gene expression assays.

Gene	TaqMan Assay ID
Bmp2	Mm01340178_m1
Bmpr1a	Mm00477650_m1
Mvh (=*Ddx4*)	Mm00802445_m1
Oct4 (=*Pou5f1*)	Mm00658129_gH
Spo11	Mm00488871_m1
Stra8	Mm00486473_m1
Sycp3	Mm00488519_m1
Tbp	Mm00446973_m1
Zglp1	Mm01178045_m1

Table S8. List of primary and secondary antibodies used.

Antibodies	Dilution	Source	RRID
Rabbit polyclonal anti-BMPR1A	1:150	38-6000, Thermo Fisher Scientific	AB_2533377
Rabbit polyclonal anti-DDX4	1:800	ab13840, Abcam	AB_443012
Mouse monoclonal anti-DDX4	1:500	ab27591, Abcam	AB_11139638
Mouse monoclonal anti-Oct-3/4	1:50	sc-5279, Santa Cruz Biotechnology	AB_628051
Rabbit polyclonal anti-phospho-Histone H3 (Ser10)	1:200	06-570, Millipore	AB_310177
Rabbit polyclonal anti-STRA8	1:200	ab49405, Abcam	AB_945677
Rabbit polyclonal anti-SYCP1	1:200	ab15090. Abcam	AB_301636
Rabbit polyclonal anti-SYCP3	1:200	ab15093, Abcam	AB_301639
Mouse monoclonal anti-SYCP3	1:200	ab97672, Abcam	AB_10678841
Mouse monoclonal anti-γH2AX	1:200	05-636, Millipore	AB_309864
Rat monoclonal anti-GCNA (TRA98)	1:500	ab82527, Abcam	AB_1659152
Mouse monoclonal anti-BrdU (Bu20a)	1:200	5292, Cell Signalling	AB_10548898
Goat Anti-Mouse IgG (H+L) Alexa Fluor 594	1:200 (paraffin section) or 1:2000 (meiotic spreads)	A11032, Invitrogen	
Goat Anti-Rabbit IgG (H+L) Alexa Fluor 488	1:200 (paraffin section) or 1:2000 (meiotic spreads)	A11034, Invitrogen	
Donkey Anti-Rabbit IgG (H+L) Alexa Fluor 594	1:200	A21207, Invitrogen	
Goat Anti-Rat IgG Alexa Fluor 488	1:200	A21204, Invitrogen	

Development • Supplementary information
